# Real-World Renal Effects of Dapagliflozin in Bulgarian Patients with Chronic Kidney Disease of Predominantly Glomerular Origin: A Two-Year Comparative Cohort Study with Historical Controls

**DOI:** 10.3390/biomedicines14102353

**Published:** 2026-10-16

**Authors:** Veronica Aladzhova, Teodora Ilieva, Bilyana Vasileva, Mariana Petkova, Martin Milkinski, Simona Kuzmanova-Hrischeva, Simona Kerezieva, Ina Georgieva, Ivan Trendafilov, Maria Radanova, Vasil Vasilev

**Affiliations:** 1 Department of Nephrology, Medical Faculty, Medical University of Sofia, 1431 Sofia, Bulgaria; v.aladzova@medfac.mu-sofia.bg (V.A.); t.ilieva@medfac.mu-sofia.bg (T.I.); b.vasileva@medfac.mu-sofia.bg (B.V.); m.petkova@medfac.mu-sofia.bg (M.P.); m.milkinski@medfac.mu-sofia.bg (M.M.); shrischeva@medfac.mu-sofia.bg (S.K.-H.); s.kerezieva@medfac.mu-sofia.bg (S.K.); i.georgieva@medfac.mu-sofia.bg (I.G.); i.trendafilov@medfac.mu-sofia.bg (I.T.); 2 Clinic of Nephrology, University Hospital “Tsaritsa Yoanna–ISUL”, 1527 Sofia, Bulgaria; 3 Clinic of Dialysis Treatment, University Hospital “Alexandrovska”, 1431 Sofia, Bulgaria; 4 Department of Biochemistry, Molecular Medicine and Nutrigenomics, Faculty of Pharmacy, Medical University of Varna, 9002 Varna, Bulgaria; maria.radanova@mu-varna.bg

**Keywords:** dapagliflozin, SGLT2 inhibitors, glomerular disease, proteinuria, glomerular filtration rate, immunosuppression, chronic kidney disease, real-world evidence

## Abstract

**Background/Objectives:** Sodium–glucose cotransporter 2 (SGLT2) inhibitors are established in the treatment of chronic kidney disease (CKD), but evidence in CKD caused by glomerular disease is limited, and patients on intensified immunosuppression have been excluded from the randomised trials. We examined the effect of dapagliflozin on proteinuria and kidney function over two years in such a population. **Methods:** In this single-centre retrospective cohort study, we compared 147 patients treated with dapagliflozin 10 mg daily between 2021 and 2025 with 170 historical controls followed between 2014 and 2021; most had biopsy-proven glomerular diseases. Proteinuria and estimated glomerular filtration rate (eGFR) were assessed at months 0, 12 and 24, and comparisons used analysis of covariance adjusted for baseline. Subgroups on intensified immunosuppression at entry were prespecified. **Results:** Proteinuria fell by 51.9% under dapagliflozin and by 22.2% in the historical cohort; the adjusted geometric mean ratio (GMR) was 0.684 (95% CI 0.534–0.878; *p* = 0.003). During the second year, eGFR declined more slowly with dapagliflozin (a difference of 4.61 mL/min/1.73 m^2^ per year; 95% CI 1.82–7.41; *p* = 0.001), although eGFR at month 24 did not differ significantly after adjustment for baseline (*p* = 0.15). An eGFR below 15 mL/min/1.73 m^2^ was reached by 0.7% of treated patients and by 11.8% of controls. Among patients on intensified immunosuppression, proteinuria fell by 67.5% when dapagliflozin was added, compared with 4.6% on the same treatment alone (adjusted GMR 0.371; 95% CI 0.234–0.586; *p* < 0.001). **Conclusions:** Dapagliflozin was associated with a progressive antiproteinuric effect, most marked in patients on intensified immunosuppression, and with a slower decline in kidney function during the second year. Because this study was observational, the true effects are likely to be smaller than those observed.

## 1. Introduction

Chronic kidney disease (CKD) affects about 788 million adults worldwide, 14.2% of the adult population, and in 2023 it was the ninth leading cause of death [1]. Sodium–glucose cotransporter 2 (SGLT2) inhibitors are the first drug class since renin–angiotensin system blockade with angiotensin-converting enzyme inhibitors (ACEi) and angiotensin receptor blockers (ARB) to substantially change the treatment of CKD. In the DAPA-CKD trial, dapagliflozin reduced a composite of kidney disease progression and death by 39% [2], and EMPA-KIDNEY confirmed the benefit of empagliflozin in a broader population, including patients with an estimated glomerular filtration rate (eGFR) as low as 20 mL/min/1.73 m^2^ and those without albuminuria [3]. A meta-analysis of the large placebo-controlled trials of SGLT2 inhibitors confirmed that the kidney benefit is largely independent of diabetes status [4]. On this evidence, the KDIGO 2024 guideline recommends an SGLT2 inhibitor for most adults with chronic kidney disease, alongside renin–angiotensin system blockade [5].

The mechanism is only partly haemodynamic: inhibition of proximal sodium reabsorption restores tubuloglomerular feedback and relieves glomerular hyperfiltration, but the class also alters tubular energy handling, oxygen consumption and inflammation, and the relative weight of these pathways remains debated [6,7]. The haemodynamic component produces a characteristic early dip in eGFR followed by a slower long-term decline; this dip is functional and reversible, and separating it from the subsequent chronic slope has become standard in the analysis of these trials [8]. Albuminuria also falls, but a prespecified analysis of DAPA-CKD found that the reduction in albuminuria differed between patients with and without diabetes, whereas the effect on clinical outcomes did not. This suggests that part of the protection is mediated by pathways independent of albuminuria [9].

Evidence in CKD caused by glomerular disease is more limited. Patients with glomerulopathies were a minority in the pivotal trials; the largest single group, the 270 participants with IgA nephropathy in DAPA-CKD, showed a 71% reduction in a composite of kidney disease progression and death [10]. The data remain sparse for membranous nephropathy, focal segmental glomerulosclerosis and the proliferative glomerulonephritides, and the corresponding chapters of the KDIGO guideline have not been revised since 2021 [11]. For IgA nephropathy and lupus nephritis, the updated KDIGO guidelines of 2025 and 2024 now include SGLT2 inhibitors in supportive care [12,13]. The evidence using proteinuria as the outcome is limited and conflicting: the DIAMOND trial found no change in 24 h proteinuria after six weeks of dapagliflozin [14], while a small study of empagliflozin reported a reduction [15].

A further gap concerns patients whose disease is actively treated. The largest observational cohort confined to non-diabetic glomerulopathies excluded patients on induction immunosuppression, defined as prednisone above 20 mg daily, rituximab or cyclophosphamide within the preceding six months, although stable maintenance treatment was allowed [16]. These patients were also absent from the large randomised outcome trials. The lack of data on patients receiving background immunosuppression was pointed out several years ago [17], and a recent review concluded that the benefit probably extends to these patients but that the data are lacking [18]. The agents used to treat these diseases act directly on the immune injury. Corticosteroids suppress the transcription of pro-inflammatory cytokines and restrain lymphocyte activation, and in the podocytopathies also act directly on the podocyte. Cyclophosphamide, an alkylating agent, depletes proliferating B and T lymphocytes, while mycophenolate mofetil and azathioprine interfere with purine synthesis, on which activated lymphocytes depend. Ciclosporin, a calcineurin inhibitor, blocks T-cell activation and stabilises the podocyte cytoskeleton, but also constricts the afferent arteriole, and intravenous immunoglobulin modulates Fc-receptor signalling and complement activation [11]. SGLT2 inhibitors, by contrast, act on the haemodynamic and metabolic consequences of this injury, so the two approaches may be expected to complement rather than duplicate each other. It is therefore unknown whether an antiproteinuric effect can be demonstrated in patients receiving intensified immunosuppression, and whether it differs from that seen in quiescent disease.

Real-world evidence is unevenly distributed and particularly scarce from Eastern Europe [19,20]. Few cohorts follow the full two-year trajectory of both proteinuria and eGFR in a population defined by biopsy rather than by an albuminuria threshold.

We examined the effect of dapagliflozin on proteinuria and kidney function over two years in a Bulgarian cohort with predominantly biopsy-proven glomerular disease, compared with a historical cohort from the same centre treated before SGLT2 inhibitors entered routine clinical practice. We asked three questions: whether dapagliflozin reduces proteinuria and slows the decline in eGFR; whether any such effect persists after adjustment for baseline values and blood pressure; and whether it differs in patients on intensified immunosuppression. Blood pressure and peripheral oedema were examined as secondary outcomes. Blood pressure was included because a fall in blood pressure could by itself lower proteinuria and had to be excluded as an explanation; oedema was included to assess fluid balance, given the natriuretic action of dapagliflozin. Patients whose disease requires intensified immunosuppression are the group about whom the least is known; they were excluded from the outcome trials and from the larger observational cohorts, and the randomised studies in lupus nephritis have concerned inactive or long-standing disease [21,22]. A comparison of such patients with others on the same treatment without dapagliflozin has not, as far as we are aware, been reported.

## 2. Materials and Methods

### 2.1. Design, Setting and Participants

We conducted a single-centre retrospective cohort study with historical controls at the Clinic of Nephrology, University Hospital “Tsaritsa Yoanna–ISUL”, Sofia; the dapagliflozin cohort was treated and followed between September 2021 and December 2025. The historical cohort was followed at the same clinic between February 2014 and September 2021, before SGLT2 inhibitors entered routine practice. The periods do not overlap, and no patient appears in both cohorts.

Of 301 patients who started dapagliflozin, 147 were analysed (Figure S1). The largest excluded group, comprising 125 patients, had started treatment too late to complete 24 months of follow-up by the end of December 2025. A further 29 were excluded for other reasons: 10 did not attend a scheduled admission, seven were diagnosed with a malignancy during follow-up, and four withdrew consent for the use of their clinical data (one at the first admission and three at month 12), and none of their data were used. Five stopped treatment of their own accord, and in three it was withdrawn for recurrent urinary tract infection. The historical cohort was drawn from the records of 502 patients seen between February 2014 and September 2021. Of these, 298 did not have three admissions at the required time points, 14 did not attend a scheduled admission, and 20 reached end-stage kidney disease before month 24. The remaining 170 met the same criteria as the dapagliflozin cohort and were all included; no further selection was applied.

Patients were eligible if they were aged 18 or over, had CKD by the KDIGO 2024 criteria and an eGFR of at least 15 mL/min/1.73 m^2^ at baseline and had complete data from three admissions for scheduled review of their glomerular disease (at baseline and at approximately 12 and 24 months, each within one month). In the dapagliflozin cohort, treatment was initiated only at an eGFR of at least 25 mL/min/1.73 m^2^, in line with the prescribing information in force at the time and could then be continued down to 15 mL/min/1.73 m^2^. In 10 patients, dapagliflozin had been started on the basis of an eGFR of at least 25 mL/min/1.73 m^2^, measured within the month before the baseline admission; at the baseline admission, their eGFR was slightly below this threshold (range 20.3–24.5 mL/min/1.73 m^2^). In the remaining 137, dapagliflozin was started at the baseline admission. CKD was defined as abnormalities of kidney structure or function persisting for more than three months, that is, an eGFR below 60 mL/min/1.73 m^2^ or a marker of kidney damage (proteinuria, urine sediment abnormalities, imaging or histological abnormalities) and was staged by eGFR. All participants therefore had established CKD; patients with glomerular disease who did not meet these criteria were not eligible. In eight patients whose eGFR and proteinuria were normal at baseline, the criterion was met by histological abnormalities. The underlying cause was a glomerular disease in about two thirds of the patients (98 of 147 in the dapagliflozin cohort and 122 of 170 in the historical cohort); the remainder had chronic interstitial nephritis, diabetic or hypertensive nephropathy as the only histological finding, or no histological diagnosis (Table 1). All patients were required to have given the standard written consent for the use of their clinical data for scientific purposes; four patients in the dapagliflozin cohort withdrew consent and were not included. Exclusion criteria were acute kidney injury on CKD, active infection of any kind, including urinary tract infection at the time of sampling at any visit, obstructive uropathy, terminal malignancy and an eGFR below 15 mL/min/1.73 m^2^. Refusal of consent for the use of clinical data was a further ground for exclusion. No patient included in the analysis was receiving dialysis at entry or during follow-up; an eGFR below 15 mL/min/1.73 m^2^, used as an outcome, marks progression to kidney failure rather than the start of kidney replacement therapy.

### 2.2. Assessments

Patients were admitted at baseline and at 12 and 24 months for history, examination and laboratory tests. Recorded variables were age, sex, diagnosis and its histological confirmation, coexisting diabetes and hypertension, concomitant medication, blood pressure and peripheral oedema, a standard biochemical and haematological panel (Table 1), and 24 h urinary protein. Blood pressure was measured according to the standardised office protocol recommended by KDIGO [23], and mean arterial pressure was calculated as diastolic pressure plus one third of the pulse pressure. All renal biopsies were reported by a single pathologist. Records were extracted by investigators who took no part in the analysis.

All samples from both cohorts were analysed in the hospital laboratory, on the same analysers and by the same methods throughout 2014–2025. Serum creatinine was measured by a standardised, IDMS-traceable compensated Jaffe (alkaline picrate) method on an Abbott Architect c8000 analyser (Abbott Laboratories, Abbott Park, IL, USA), and eGFR was derived from the race-free 2021 CKD-EPI equation [24]. The same analyser was used for urea (urease–glutamate dehydrogenase), uric acid (uricase), glucose (hexokinase), total protein (biuret), total cholesterol (cholesterol oxidase) and triglycerides (glycerol phosphate oxidase), as well as for sodium, potassium and chloride, which were measured with indirect ion-selective electrodes. Serum albumin was measured by the bromocresol green method on a BioSystems A25 analyser (BioSystems, Barcelona, Spain). Protein in 24 h urine collections was measured by the pyrogallol red–molybdate method on a Siemens Dimension EXL analyser (Siemens Healthineers, Erlangen, Germany) and is given in g/24 h. Haemoglobin and haematocrit were determined as part of the full blood count on Siemens Advia 2120 and Advia 560 haematology analysers (Siemens Healthineers, Erlangen, Germany).

### 2.3. Treatment of Glomerular Diseases and Definition of Intensified Immunosuppression

Glomerular disease was treated according to the KDIGO recommendations in force at the time: the 2012 guideline for the historical cohort [25] and its 2021 revision with subsequent updates for the dapagliflozin cohort [11,12,13]. Treatment included corticosteroids, cyclophosphamide, azathioprine, mycophenolate mofetil, ciclosporin or intravenous immunoglobulin, alone or in combination, as the diagnosis required. Not all patients received immunosuppression. Intensified immunosuppression at entry was given to 41 of 147 patients (27.9%) in the dapagliflozin cohort and 57 of 170 (33.5%) in the historical cohort, and about half of each cohort received intensified immunosuppression at one or more of the three visits. Immunosuppression also did not always imply an immune-mediated glomerulopathy. Of the 98 patients on intensified treatment at entry, 77 had an immune-mediated glomerular disease and 8 chronic interstitial nephritis; 11 were treated empirically without a histological diagnosis, and in 2, corticosteroids were started on the suspicion of an immune-mediated disease before a biopsy that showed diabetic or hypertensive nephropathy. This study therefore compares dapagliflozin added to standard care with standard care alone, not dapagliflozin with immunosuppression. The same immunosuppressive agents were available and used in both periods, although in somewhat different proportions (Table S2), and rituximab was given to no patient, as it is not yet available to nephrologists in Bulgaria for glomerular disease. Background treatment was also similar wherever it could be measured: renin–angiotensin system blockade, mineralocorticoid receptor antagonists, diuretics and intensified immunosuppression were used in comparable proportions in the two cohorts (Table 1). All patients in the dapagliflozin cohort received 10 mg once daily, taken in the morning; this dose was used from the start of treatment and was not titrated, and no temporary interruption of treatment was recorded in any patient. Dapagliflozin was added to existing treatment: in patients receiving an ACEi or ARB, these were continued at the highest tolerated dose, and diuretic doses were adjusted where the clinical situation required it.

Subgroups were specified in advance. In each cohort, the subgroup comprised patients whose disease was active enough at entry to require intensified immunosuppression, defined as treatment at any point in the preceding three months with a corticosteroid at a dose equivalent to at least 20 mg prednisolone daily, or with azathioprine, mycophenolate mofetil, cyclophosphamide, ciclosporin or intravenous immunoglobulin at standard doses, alone or in combination. Each of these agents at standard dose represents immunosuppression at least as intense as the corticosteroid threshold, so the definition marks clinically active disease rather than any particular regimen. The agents used and the cumulative duration of treatment with each, up to every visit, were extracted retrospectively from the medical records. In 15 patients, the recorded durations were inconsistent between visits; these patients were retained in all analyses, and analyses using the duration at baseline were repeated without the five in whom it was affected.

Subgroup membership was fixed at baseline: patients who met the criterion at entry were followed to month 24 regardless of later changes in their treatment, and patients who did not meet it at entry were not added later. This approach was chosen for two reasons. First, immunosuppression is intensified in response to rising proteinuria, so a subgroup redefined at each visit would have been selected partly on the outcome under study. Second, because treatment changed during follow-up, assignment at each visit would have produced a different group of patients at each time point.

### 2.4. Ethics

The Ethics Committee of University Hospital “Tsaritsa Yoanna–ISUL” approved this study (protocol 002, approved 9 September 2026), which was conducted in accordance with the Declaration of Helsinki (2013 revision). All patients had given written consent on admission for the use of their clinical data for scientific purposes. The Ethics Committee waived the requirement for separate study-specific consent because the analysis was based entirely on existing, anonymised records. Data were anonymised in both cohorts before analysis.

### 2.5. Statistical Analysis

Normality was assessed graphically and by the Shapiro–Wilk test. Skewed variables are given as medians (IQR), additive quantities as means (95% CI) and categorical variables as *n* (%). Baseline comparisons used the Mann–Whitney U, χ^2^ or Fisher’s exact test; all tests were two-sided, with *p* < 0.05 taken as significant. Data were complete at every visit, so nothing was imputed. No sample size calculation was performed because the cohorts included every eligible patient in the two periods. No matching was applied; comparability was addressed by analysis of covariance. Instead, the effect sizes that this study could detect were estimated from the available data. With 80% power at a two-sided α of 0.05, the two cohorts were large enough to detect a 30% difference in proteinuria at 24 months (the threshold accepted as a surrogate endpoint in glomerular disease) and a difference of about 4 mL/min/1.73 m^2^ per year in the chronic eGFR slope. The subgroups were smaller: in patients on intensified immunosuppression, only a difference in proteinuria of about 48% could be detected with the same power, and in those without it one of about 34%, so that smaller effects in these groups cannot be ruled out.

Because proteinuria was strongly right-skewed, it was log-transformed, and effects were expressed as geometric mean ratios (GMRs) with 95% CI. Within-cohort changes were tested with the Friedman test, followed by pairwise Wilcoxon signed-rank tests on log values with Bonferroni correction for three comparisons. eGFR was analysed untransformed because its changes are additive; changes are given as means (95% CI) and medians (IQR), and their significance was tested with the same rank-based tests. Confidence intervals from the covariance models are based on the t distribution, in keeping with their *p*-values. For unadjusted differences between the cohorts in mean change, confidence intervals were calculated with Welch’s correction for unequal variances, whereas the *p*-values came from the Mann–Whitney U test, given the skewed distribution of the changes; near the threshold of significance the two can therefore occasionally disagree. Rates of change in eGFR were annualised for the whole period, the first year and the second year; the second-year rate served as the principal measure of chronic decline, since an early haemodynamic dip, if present, is reversible and would otherwise offset the treatment effect.

Because the cohorts began at different levels of both outcomes, and regression to the mean favours the cohort that begins higher, the principal comparisons were made by analysis of covariance: log proteinuria at month 24 was modelled on log baseline proteinuria, and the change in eGFR over the second year on baseline eGFR and, in a further model, on month-12 eGFR. Blood pressure was analysed in the same way, and sensitivity analyses added mean arterial pressure at baseline and month 24 to both outcome models. Slopes were homogeneous throughout. To examine whether the baseline imbalance accounted for the findings, the principal models were also refitted with baseline eGFR (for proteinuria) or log baseline proteinuria (for eGFR), age and diabetes as additional covariates, and repeated in the patients with a baseline eGFR of at least 25 mL/min/1.73 m^2^. A fuller model added sex, hypertension, diagnostic category, renin–angiotensin system blockade, mineralocorticoid receptor antagonists, loop and thiazide diuretics, baseline mean arterial pressure and intensified immunosuppression at entry, and in a further model immunosuppressive status at months 12 and 24. As an alternative approach, the cohorts were balanced by inverse probability of treatment weighting, using a propensity score estimated from the same baseline variables together with baseline eGFR and proteinuria, with robust standard errors. In the subgroup on intensified immunosuppression, the models were further adjusted for the duration of immunosuppression before baseline (log-transformed) and for the use of cyclophosphamide and azathioprine and repeated separately in patients treated for three months or less and in those treated for longer.

Categorical outcomes were prespecified. For proteinuria: reductions ≥30% and ≥50%, complete remission (<0.3 g/24 h), partial remission (<3.5 g/24 h with ≥50% reduction) and progression (≥30% increase); for eGFR, declines ≥30% and ≥40%, eGFR <15 mL/min/1.73 m^2^, rapid decline (>5 mL/min/1.73 m^2^ per year) and a classification of eGFR trajectories into three categories, with a change within ±10% considered stable, as this corresponds to the usual biological and analytical variation. These outcomes were compared with Fisher’s exact test, with odds ratios and 95% CI. Within-cohort change in the prevalence of peripheral oedema was assessed with the exact McNemar test, and the association between changes in blood pressure and in proteinuria with the Spearman rank correlation coefficient.

Subgroup comparisons are reported with the corresponding interaction term and with a supporting model containing diagnostic category. Subgroup and stratified analyses were exploratory and carry no multiplicity adjustment beyond the Bonferroni correction described above. Reporting follows STROBE [26].

Analyses were performed with GraphPad Prism 5.0 (GraphPad Software, San Diego, CA, USA) and Python 3.12 (Python Software Foundation, Wilmington, DE, USA) with the SciPy (version 1.17) and statsmodels (version 0.15) libraries; the covariance models and interaction tests were run in Python, being unavailable in Prism 5.0.

During the preparation of this manuscript, the authors used Claude (Claude Opus 5.5; Anthropic, San Francisco, CA, USA; accessed August–October 2026) to assist with statistical analysis and the preparation of figures. The authors have reviewed and edited the output and take full responsibility for the content of this publication.

## 3. Results

Baseline characteristics are given in Table 1. The dapagliflozin cohort was older (median 53 versus 47 years; *p* = 0.001) and had more advanced kidney disease (median eGFR 50.0 versus 67.0 mL/min/1.73 m^2^; *p* = 0.001), with more hypertension (91.2% versus 80.6%) and diabetes (27.2% versus 17.6%). Biopsy-proven immune nephropathy was present in 77.5% and 79.4% respectively.

### 3.1. Proteinuria

Proteinuria was measured at entry and at months 12 and 24 in all 317 patients, with no missing values. In the dapagliflozin cohort, proteinuria continued to fall during the second year, whereas in the historical cohort it changed little after month 12; the same pattern, more pronounced, was seen in patients on intensified immunosuppression.

#### 3.1.1. Proteinuria in All Patients 

Median baseline proteinuria was 0.95 g/24 h (IQR 0.38–2.41) under dapagliflozin and 0.62 g/24 h (IQR 0.24–1.94) in the historical cohort (*p* = 0.078). Table 2 summarises the subsequent course.

Under dapagliflozin, proteinuria fell at every interval (Friedman χ^2^ = 66.4, *p* < 0.001): by 33.8% at month 12 (GMR 0.662, 95% CI 0.560–0.781) and by 51.9% at month 24 (GMR 0.481, 95% CI 0.399–0.579), with a further 27.4% accruing during the second year alone (GMR 0.726, 95% CI 0.629–0.839). The effect accumulated rather than plateauing after an initial response.

**Figure 1:**
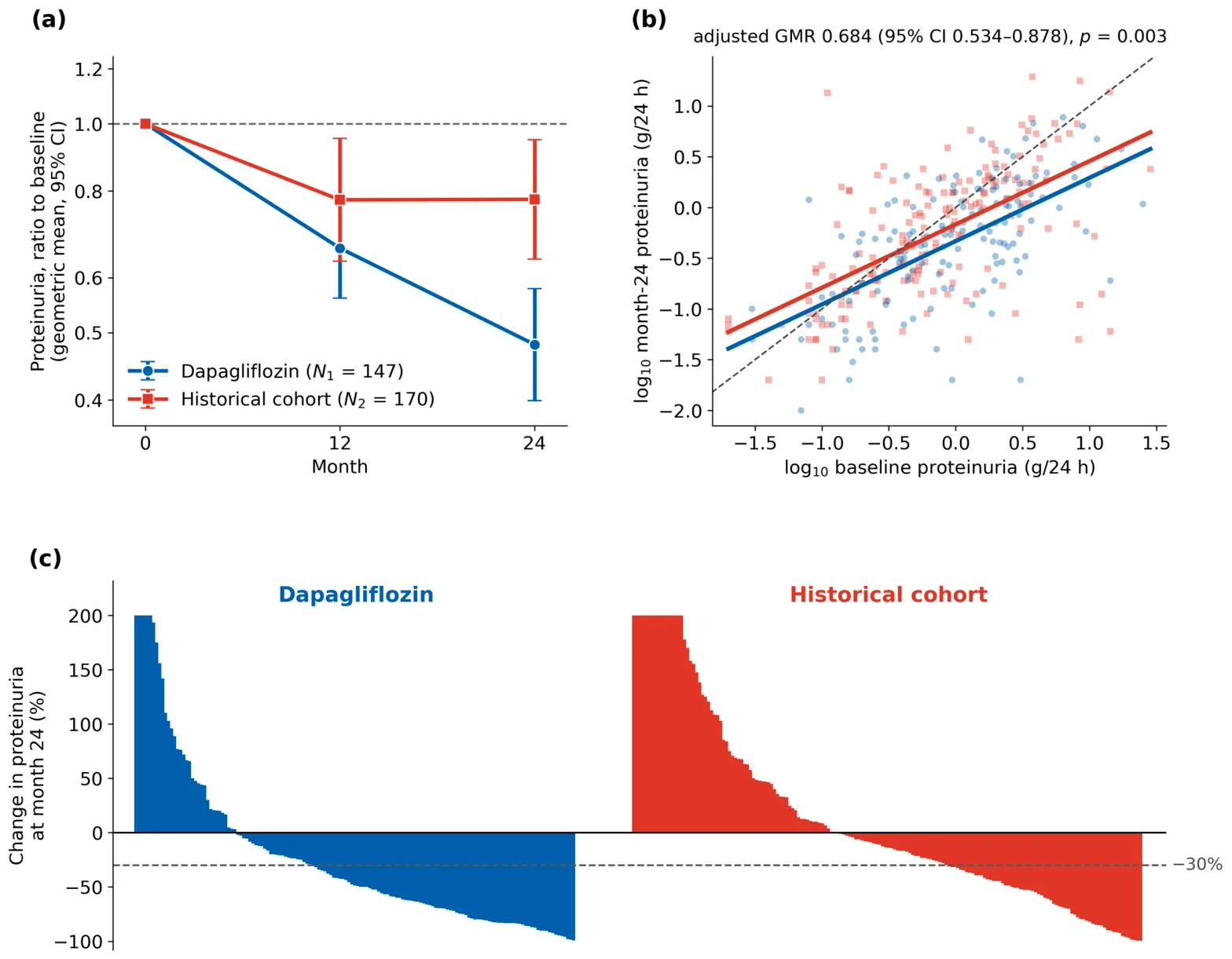
Proteinuria over 24 months in the dapagliflozin and historical cohorts. (**a**) Proteinuria expressed as the ratio to the individual baseline value (geometric mean with 95% confidence interval), plotted on a logarithmic scale; the dashed line marks the absence of change. (**b**) Analysis of covariance of log-transformed month-24 proteinuria on log-transformed baseline proteinuria, both in g/24 h; individual patients are shown as points and the fitted parallel regression lines as solid lines, the dashed diagonal being the line of identity. (**c**) Percentage change in proteinuria at month 24 for every patient, sorted in descending order within each cohort; the dashed line marks the −30% response threshold and bars are truncated at +200%. Blue, dapagliflozin cohort (N_1_ = 147); red, historical cohort (N_2_ = 170). GMR, geometric mean ratio; CI, confidence interval.

The course in the historical cohort was different. The whole of its change occurred during the first year, when proteinuria fell by 22.3% (GMR 0.777, 95% CI 0.633–0.953; adjusted *p* = 0.013); thereafter, it plateaued (GMR for months 12 to 24, 1.002, 95% CI 0.835–1.202), and the cumulative 24-month reduction did not survive correction for multiple comparisons (GMR 0.778, 95% CI 0.638–0.948; adjusted *p* = 0.063). Figure 1a shows the diverging trajectories. Compared directly, the two cohorts did not differ in proteinuria at any single visit (*p* = 0.078, 0.464 and 0.323 at months 0, 12 and 24). This is largely because the dapagliflozin cohort started higher, drew level with the historical cohort at month 12 and ended below it, and a comparison of raw values at one visit takes no account of where each patient began. With baseline taken into account, the cohorts were indistinguishable at month 12 (adjusted GMR 0.940, 95% CI 0.737–1.201; *p* = 0.621) and clearly separated by month 24 (0.684, 95% CI 0.534–0.878; *p* = 0.003) (Table 2).

Comparing the individual changes directly, the cohorts did not differ at month 12 (ratio of GMRs 0.852, 95% CI 0.655–1.107; *p* = 0.064) but did so markedly at month 24 (0.618, 95% CI 0.471–0.810; *p* < 0.001).

Regression to the mean proved considerable: the coefficient on baseline proteinuria was 0.624 (95% CI 0.534–0.713); without regression to the mean, it would have been close to unity. Regression to the mean accounted for only part of the difference. With baseline held constant, month-24 proteinuria was 31.6% lower under dapagliflozin (adjusted GMR 0.684, 95% CI 0.534–0.878; *p* = 0.003; homogeneous slopes, interaction *p* = 0.574), and adjustment for blood pressure barely altered this (Section 3.3). Further adjustment for baseline eGFR, age and diabetes left the estimate essentially unchanged (adjusted GMR 0.679, 95% CI 0.527–0.876; *p* = 0.003), as did restriction to patients with a baseline eGFR of at least 25 mL/min/1.73 m^2^ (0.710, 95% CI 0.546–0.922; *p* = 0.010). In the fully adjusted model, the estimate was 0.664 (95% CI 0.514–0.857; *p* = 0.002), and after inverse probability weighting, which balanced all baseline characteristics (standardised differences below 0.05), it was 0.616 (0.465–0.816; *p* < 0.001) (Table S1). Figure 1b shows the fitted lines with the individual observations.

Responder rates are given in Table 3. A reduction of at least 30%, a validated surrogate endpoint in glomerular disease, was reached by 59.2% under dapagliflozin against 38.2% of the historical cohort (OR 2.34, 95% CI 1.49–3.68; *p* < 0.001), and a reduction of at least 50% by 48.3% against 25.9% (OR 2.68, 95% CI 1.67–4.29). Progression was less frequent with dapagliflozin, occurring in 17.0% against 30.6% (OR 0.47, 95% CI 0.27–0.80; *p* = 0.006). Complete remission was the one outcome that did not favour dapagliflozin (33.3% against 40.6%; OR 0.73, 95% CI 0.46–1.16; *p* = 0.20), because the lower baseline proteinuria of the historical cohort placed it closer to the threshold. Figure 1c shows the individual responses.

An exploratory analysis stratified by baseline proteinuria showed a consistent difference across the subnephrotic range: below 0.5 g/24 h, the dapagliflozin cohort improved while the historical cohort worsened (GMR 0.784 versus 1.245; *p* = 0.047), with the same ordering at 0.5–1.0 g/24 h (0.474 versus 0.849; *p* = 0.074) and 1.0–3.5 g/24 h (0.368 versus 0.611; *p* = 0.010). Above 3.5 g/24 h, both improved almost identically (0.265 versus 0.261; *p* = 0.39). Only 19 and 24 patients fell into this uppermost stratum, and intensified immunosuppression was concentrated within it, so baseline proteinuria alone gives an incomplete account of who responded; treatment status at entry is examined next.

#### 3.1.2. Proteinuria in Patients Receiving Intensified Immunosuppression at Baseline

Forty-one patients under dapagliflozin and 57 in the historical cohort were on intensified immunosuppression at entry. Baseline proteinuria was again numerically higher under dapagliflozin without reaching significance (1.14 g/24 h, IQR 0.28–2.35 versus 0.59 g/24 h, IQR 0.25–2.88; *p* = 0.41).

Since these subgroups were defined at entry, treatment could and did change thereafter. Under dapagliflozin, 27 of the 41 patients (65.9%) still met the criterion for intensified immunosuppression at month 12 and 21 (51.2%) at month 24; in the historical cohort, the figures were closely similar, 38 of 57 (66.7%) and 31 (54.4%). Neither difference approached significance (*p* = 1.00 and *p* = 0.84), so exposure to intensified immunosuppression was comparable, and the divergence described below cannot be attributed to a differential rate of immunosuppression withdrawal. In the cohorts as a whole, intensified immunosuppression was received at months 0, 12 and 24 by 27.9%, 36.1% and 26.5% of patients on dapagliflozin and by 33.5%, 35.3% and 26.5% of historical patients (*p* = 0.33, 0.91 and 1.00). Among patients who were not on intensified treatment at entry, it was started during follow-up in 31 of 106 (29.2%) and 24 of 113 (21.2%), respectively (*p* = 0.21). The agents used and the duration of treatment before baseline are shown in Table S2. Cyclophosphamide was used somewhat more often under dapagliflozin and azathioprine less often (46.3% against 29.8%, *p* = 0.14, and 4.9% against 22.8%, *p* = 0.021), and patients on dapagliflozin had been treated for a shorter time (median 3 months, IQR 2–5, against 7, IQR 3–12; *p* = 0.025). Adjusting for the duration of immunosuppression and the agents used left the estimate for the subgroup essentially unchanged (0.364, 95% CI 0.223–0.591; *p* < 0.001), as did excluding the five patients whose baseline duration was uncertain. The effect was present both in patients treated for three months or less (0.263, 95% CI 0.110–0.625; *p* = 0.003) and in those treated for longer (0.502, 0.298–0.845; *p* = 0.010), with no significant interaction (*p* = 0.17).

**Figure 2:**
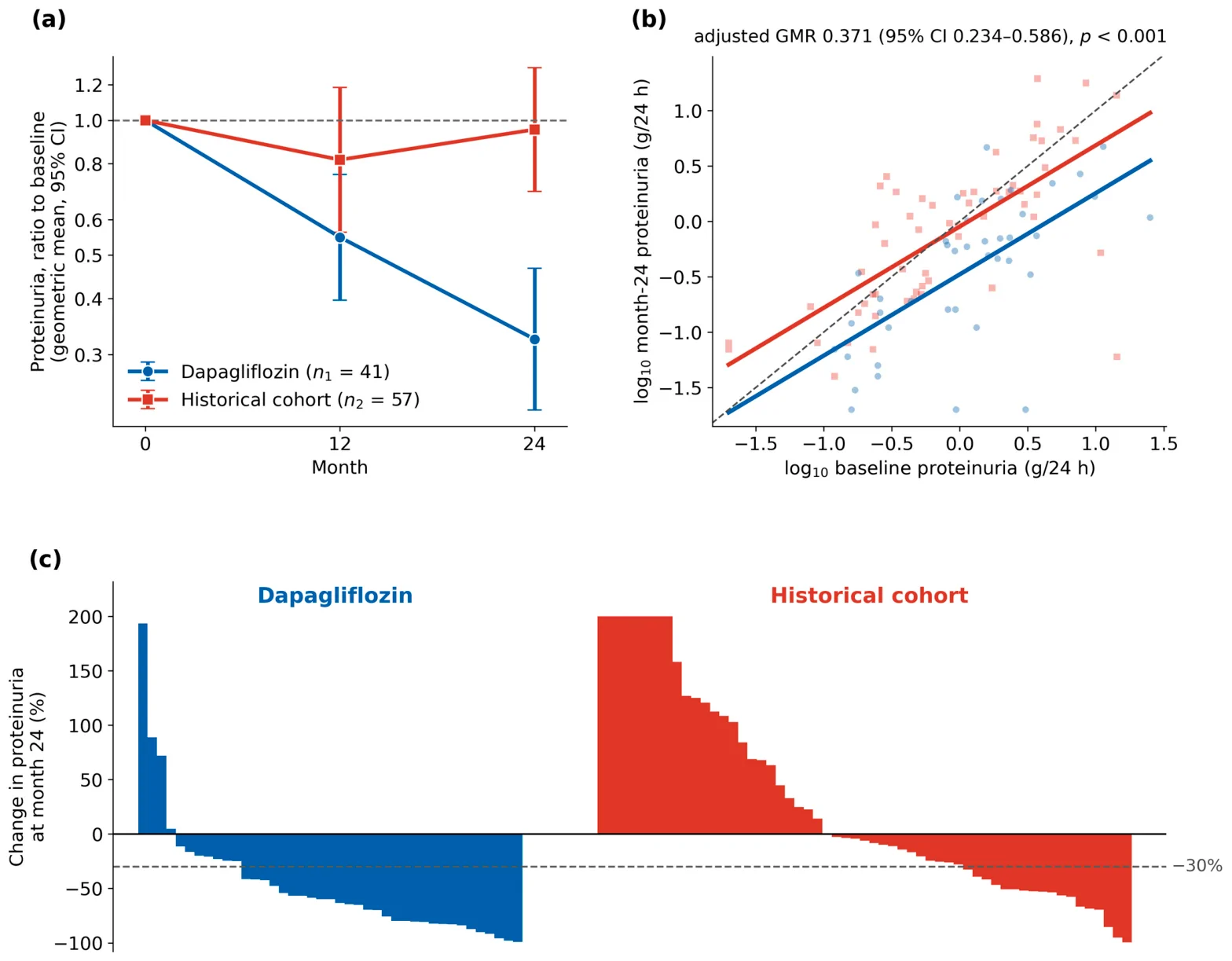
Proteinuria over 24 months in patients receiving intensified immunosuppressive therapy at baseline. (**a**) Proteinuria expressed as the ratio to the individual baseline value (geometric mean with 95% confidence interval) on a logarithmic scale; the dashed line marks the absence of change. (**b**) Analysis of covariance of log-transformed month-24 proteinuria on log-transformed baseline proteinuria, both in g/24 h; individual patients are shown as points and the fitted parallel regression lines as solid lines, the dashed diagonal being the line of identity. (**c**) Percentage change in proteinuria at month 24 for every patient, sorted in descending order within each cohort; the dashed line marks the −30% response threshold and bars are truncated at +200%. Blue, dapagliflozin cohort (n_1_ = 41); red, historical cohort (n_2_ = 57). GMR, geometric mean ratio; CI, confidence interval.

In this subgroup, the difference between the cohorts was more pronounced than in the full analysis (Table 4, Figure 2a). Under dapagliflozin, proteinuria fell by 45.2% in the first year (GMR 0.548, 95% CI 0.397–0.757) and by 67.5% by month 24 (GMR 0.325, 95% CI 0.226–0.467; Friedman χ^2^ = 36.5, *p* < 0.001), with a further 40.8% during the second year alone (GMR 0.592, 95% CI 0.446–0.785). The historical cohort did not improve.

Proteinuria in the historical cohort changed little in the first year (GMR 0.816, 95% CI 0.562–1.185; adjusted *p* = 0.48) and then drifted upwards (GMR for months 12 to 24, 1.168, 95% CI 0.825–1.654), leaving a cumulative change of 4.6% (GMR 0.954, 95% CI 0.694–1.310; Friedman χ^2^ = 4.71, *p* = 0.10). It did not fall further over the two years despite continuing immunosuppression and renin–angiotensin system blockade.

The cohorts were not clearly separated at month 12 (ratio of GMRs 0.672, 95% CI 0.413–1.093; *p* = 0.055) but were markedly so by month 24 (0.340, 95% CI 0.211–0.548; *p* < 0.001). Adjusted for baseline, month-24 proteinuria was 62.9% lower under dapagliflozin (adjusted GMR 0.371, 95% CI 0.234–0.586; *p* < 0.001; slopes homogeneous, interaction *p* = 0.66) (Figure 2b). With baseline eGFR, age and diabetes added to the model, the estimate was 0.407 (95% CI 0.252–0.657; *p* < 0.001), and the interaction with the subgroup remained significant (*p* = 0.006). The fully adjusted estimate was 0.335 (95% CI 0.198–0.566; *p* < 0.001) and the weighted estimate 0.350 (0.215–0.570; *p* < 0.001); adding immunosuppressive status at months 12 and 24 made no material difference (Table S1).

Responder rates followed the same pattern (Table 5, Figure 2c). A reduction of at least 30% was achieved by 73.2% under dapagliflozin against 31.6% in the historical cohort (OR 5.91, 95% CI 2.43–14.37; *p* < 0.001), while progression occurred in 7.3% against 36.8% (OR 0.14, 95% CI 0.04–0.49; *p* < 0.001). Complete remission again did not separate the cohorts (39.0% versus 35.1%; OR 1.18, 95% CI 0.52–2.72; *p* = 0.83).

Histological diagnosis was available in 39 of the 41 patients under dapagliflozin and 48 of the 57 in the historical cohort; the rest had not undergone biopsy and were treated empirically. Diagnostic composition was broadly alike, though proliferative and immune-complex nephropathies were somewhat commoner in the historical cohort and podocytopathies under dapagliflozin (Table 6; χ^2^ across the three categories, *p* = 0.21).

These entities differ in natural history, so the covariance model was repeated with diagnostic category included. It made little difference: across the 87 patients with a histological diagnosis, the adjusted GMR moved from 0.371 to 0.408 (95% CI 0.243–0.683; *p* < 0.001), leaving the divergence unexplained by diagnostic composition.

The effect was similar in direction and magnitude across all categories. In proliferative and immune-complex nephropathies, proteinuria fell by 58.9% under dapagliflozin against a slight rise in the historical cohort (GMR 0.411 versus 1.047; *p* = 0.002); in podocytopathies, it fell by 78.6% against 29.9% (0.214 versus 0.701; *p* = 0.010). Only ten patients fell into the remaining category, too few to interpret (0.406 versus 0.751; *p* = 0.76). There was no evidence of heterogeneity (interaction *p* = 1.00).

Among the 219 patients who were not on intensified immunosuppression at baseline, dapagliflozin had no detectable effect on proteinuria (adjusted GMR 0.894, 95% CI 0.667–1.198; *p* = 0.45), and the contrast with the subgroup was supported by a formal test for interaction (*p* = 0.002). The antiproteinuric effect thus appears concentrated among patients whose disease was active enough to require intensified treatment. The analysis was exploratory, unadjusted for multiplicity, and needs confirmation in an independent cohort.

### 3.2. Kidney Function

Both cohorts lost eGFR over the two years, but the timing differed: under dapagliflozin, the decline was confined to the first year, whereas in the historical cohort, it continued and accelerated during the second.

#### 3.2.1. Kidney Function in All Patients

The dapagliflozin cohort began with worse kidney function: median eGFR 50.0 mL/min/1.73 m^2^ (IQR 31.1–67.3) against 67.0 (IQR 37.9–92.0; *p* = 0.001), reflecting its higher proportion of patients in stages G3b and G4 (Table 1). Table 7 summarises the subsequent course.

**Figure 3:**
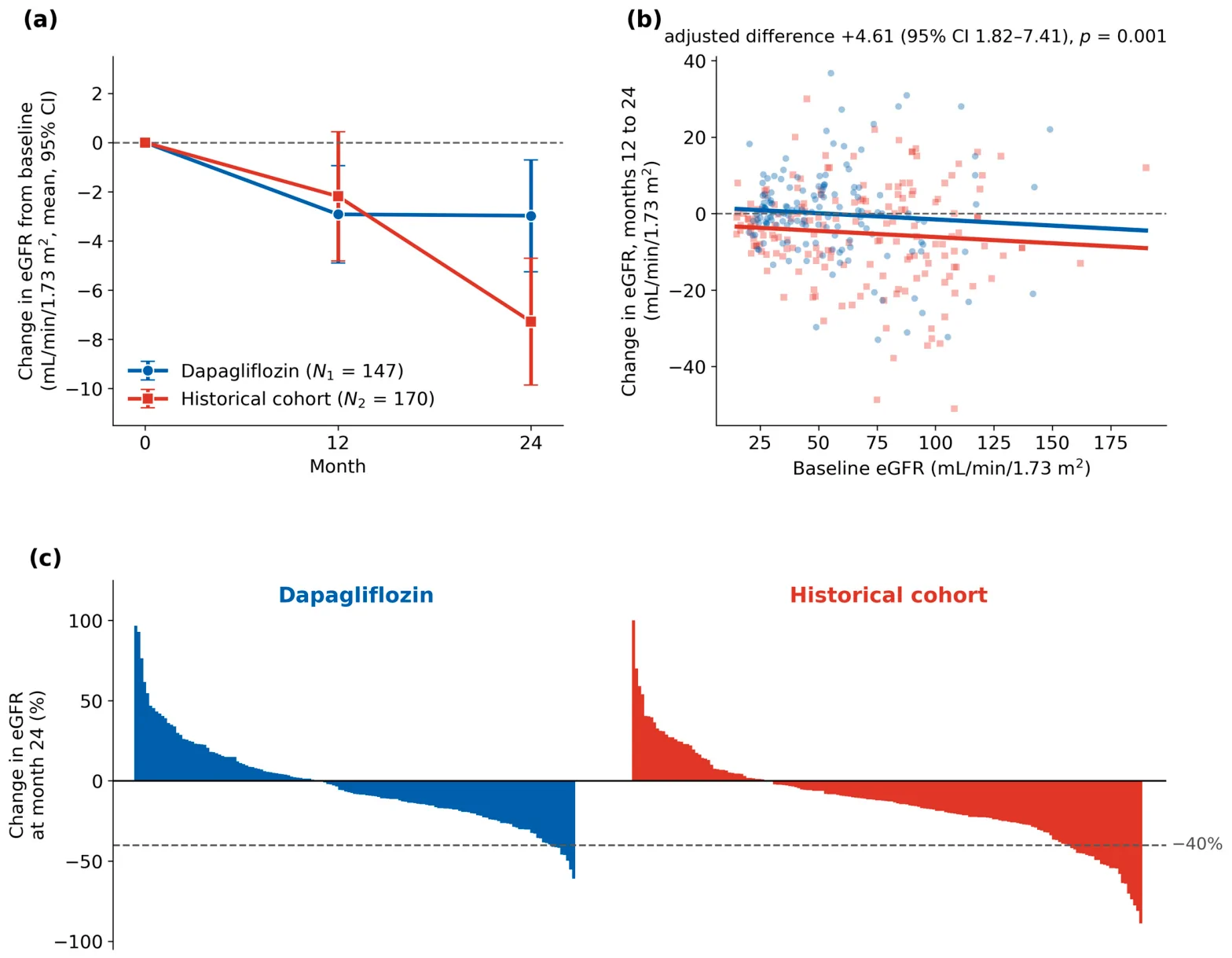
Estimated glomerular filtration rate over 24 months in the dapagliflozin and historical cohorts. (**a**) Mean change in eGFR from the individual baseline value, with 95% confidence intervals; the dashed line marks the absence of change. (**b**) Analysis of covariance of the change in eGFR between months 12 and 24 adjusted for baseline eGFR; individual patients are shown as points and the fitted parallel regression lines as solid lines, with the dashed horizontal line marking no change. (**c**) Percentage change in eGFR at month 24 for every patient, sorted in descending order within each cohort; the dashed line marks the −40% decline threshold and bars are truncated at ±100%. Blue, dapagliflozin cohort (N_1_ = 147); red, historical cohort (N_2_ = 170). CI, confidence interval.

Under dapagliflozin, eGFR fell by 2.92 mL/min/1.73 m^2^ during the first year (95% CI −4.90 to −0.94; adjusted *p* = 0.004) and then stopped falling: the change over the second year was −0.06 (95% CI −1.96 to +1.85), indistinguishable from zero. The historical cohort did the reverse—a smaller, non-significant first-year change (−2.19; 95% CI −4.81 to +0.43) followed by an accelerating decline of −5.09 over the second year (95% CI −7.05 to −3.14; adjusted *p* < 0.001). Cumulative losses were −2.98 and −7.28 mL/min/1.73 m^2^ (Figure 3a).

The first-year fall under dapagliflozin may partly reflect a haemodynamic effect, although with annual measurements this cannot be established; because such an effect would move eGFR downwards, a window spanning months 0 to 24 could understate the treatment effect, and the chronic rate of change, between months 12 and 24, was therefore taken as the principal measure.

Annualised rates bear this out. Over months 0 to 24, the decline was −1.49 mL/min/1.73 m^2^ per year under dapagliflozin against −3.64 in the historical cohort, a difference of 2.15 (95% CI 0.44–3.87; *p* = 0.005). During the first year, the cohorts were indistinguishable (−0.73; 95% CI −4.01 to +2.54; *p* = 0.56), which is compatible with an early haemodynamic dip offsetting any benefit, although annual measurements cannot show this directly. Over the second year, the difference widened to 5.04 per year (95% CI 2.32–7.76; *p* < 0.001).

Adjusting month-24 eGFR for its baseline gave a difference of 2.51 mL/min/1.73 m^2^ in favour of dapagliflozin, which did not reach significance (95% CI −0.92 to +5.93; *p* = 0.15). This was expected, because baseline eGFR alone explained most of the variation in the month-24 value (coefficient 0.864, 95% CI 0.810–0.917; R^2^ = 0.77), leaving little residual variation in which a treatment effect could be detected. Modelling the total annual rate instead gives the same result, because adjusting a follow-up value for its baseline is mathematically equivalent to adjusting the change from baseline for the same baseline.

Analysing the second year separately gave a different result. With the chronic rate as the outcome, the difference favoured dapagliflozin by 4.61 mL/min/1.73 m^2^ per year (95% CI 1.82–7.41; *p* = 0.001), with adjusted rates of −0.28 against −4.90 at the mean baseline (Figure 3b). Adding month-12 eGFR, so that both cohorts are compared from the same starting point, barely changed this (3.92 per year; 95% CI 1.34–6.51; *p* = 0.003); the second-year divergence is therefore not simply a reflection of where the cohorts stood at month 12. Further adjustment for baseline proteinuria, age and diabetes gave a difference of 4.73 mL/min/1.73 m^2^ per year (95% CI 1.89–7.57; *p* = 0.001), and restriction to patients with a baseline eGFR of at least 25 mL/min/1.73 m^2^ gave a difference of 4.91 (95% CI 1.78–8.03; *p* = 0.002). The difference in chronic slope was 4.92 mL/min/1.73 m^2^ per year (95% CI 1.97–7.86; *p* = 0.001) in the fully adjusted model and 5.43 (2.30–8.56; *p* < 0.001) after weighting, whereas the difference in eGFR at month 24 remained uncertain (2.79, 95% CI −0.72 to 6.30, and 3.70, 0.01 to 7.40, respectively; Table S1). Adjusting for blood pressure left it much the same (Section 3.3). Slopes were homogeneous throughout (all interaction *p* ≥ 0.37).

Categorical outcomes point the same way (Table 8). A decline of at least 40%, a validated surrogate for progression to kidney failure, occurred in 5.4% under dapagliflozin against 14.1% in the historical cohort (OR 0.35, 95% CI 0.15–0.81; *p* = 0.014), and rapid decline in 27.2% against 42.4% (OR 0.51, 95% CI 0.32–0.82; *p* = 0.007). The gap in eGFR below 15 mL/min/1.73 m^2^ was wider still—one patient against 20 (0.7% versus 11.8%; OR 0.05, 95% CI 0.01–0.39; *p* < 0.001), although at entry the historical cohort had more patients close to this threshold, 21 against 10 with an eGFR below 25 mL/min/1.73 m^2^.

Classifying each patient by the direction of change, and taking ±10% as stability, fewer treated patients lost more than 10% of their baseline eGFR (44.9% versus 57.1%; OR 0.61, 95% CI 0.39–0.96; *p* = 0.033) and in more of them eGFR rose by more than 10% (25.2% versus 15.9%; OR 1.78, 95% CI 1.02–3.10; *p* = 0.049); similar proportions stayed stable (29.9% versus 27.1%). Across the three categories together the difference just missed conventional significance (χ^2^ *p* = 0.053). Figure 3c shows the individual changes.

#### 3.2.2. Kidney Function in Patients Receiving Intensified Immunosuppression at Baseline

The same subgroup was examined for kidney function. Its members had better-preserved eGFR than the cohorts as a whole, as would be expected where immune disease is active but has not yet caused advanced structural damage: median 62.0 mL/min/1.73 m^2^ (IQR 37.0–90.6) under dapagliflozin and 82.1 (IQR 44.0–104.0) in the historical cohort (*p* = 0.24). Table 9 sets out the 24-month course.

The direction of change matched that of the full cohorts. Under dapagliflozin, eGFR fell by 4.68 mL/min/1.73 m^2^ in the first year (95% CI −8.63 to −0.72; adjusted *p* = 0.042) and then stopped falling, the change over the second year being +0.29 (95% CI −3.87 to +4.44), leaving a cumulative change of −4.39 (95% CI −9.71 to +0.93). The historical cohort declined throughout, losing 9.67 mL/min/1.73 m^2^ over the two years (95% CI −15.03 to −4.31), of which 6.62 came in the second year alone (95% CI −10.83 to −2.41; adjusted *p* = 0.013).

Adjusted for baseline, the chronic rate of eGFR decline differed by 6.45 mL/min/1.73 m^2^ per year in favour of dapagliflozin (95% CI 0.39–12.50; *p* = 0.037; interaction *p* = 0.70); adding month-12 eGFR, so that the cohorts were compared from the same point, left the estimate almost unchanged (5.69 per year; 95% CI −0.05 to 11.42; *p* = 0.052). Among categorical outcomes, only the most extreme separated the cohorts: no patient on dapagliflozin reached an eGFR below 15 mL/min/1.73 m^2^ at month 24, against nine of 57 in the historical cohort (0.0% versus 15.8%; *p* = 0.009). A decline of at least 40% occurred in 4.9% against 17.5% (*p* = 0.069) and rapid decline in 36.6% against 50.9% (*p* = 0.22), both favouring dapagliflozin without reaching significance.

These estimates are less precise than those from the full cohorts, and the subgroup is small. Unlike the corresponding analysis of proteinuria, however, this analysis gives no sign that the effect on eGFR depends on intensified immunosuppression: the test for interaction was negative (*p* = 0.34), and among the 219 patients who were not on such therapy, the adjusted difference was 3.91 mL/min/1.73 m^2^ per year (95% CI 0.84–6.97; *p* = 0.013). The effect on the rate of eGFR decline therefore appears comparable in patients with and without intensified immunosuppression, in contrast to its effect on proteinuria—though the analysis is exploratory and uncorrected for multiple comparisons.

### 3.3. Blood Pressure and Oedema

Blood pressure and oedema were secondary outcomes, examined for the reasons given in the Introduction: to see whether a fall in blood pressure could explain the antiproteinuric effect, and whether the natriuretic action of dapagliflozin led to volume depletion or fluid overload. The cohorts were well matched at entry, with no difference in systolic (*p* = 0.65), diastolic (*p* = 0.25) or mean arterial pressure (*p* = 0.88). Table 10 gives the subsequent course.

Blood pressure fell under dapagliflozin and did not change in the historical cohort. Systolic pressure fell by 6.31 mmHg in the first year (95% CI −9.34 to −3.29; adjusted *p* < 0.001) and by 6.54 over the two years (95% CI −9.99 to −3.10; adjusted *p* = 0.002), with no further change after month 12 (−0.23; 95% CI −3.14 to +2.68). Mean arterial pressure followed the same course, falling by 3.77 mmHg (95% CI −6.12 to −1.42; adjusted *p* = 0.017). The diastolic fall was smaller and confined to the first year (−3.57; 95% CI −5.37 to −1.77; adjusted *p* = 0.001). Nothing changed in the historical cohort at any interval (all Friedman *p* ≥ 0.28).

Adjusted for baseline, systolic pressure was lower under dapagliflozin at both visits—by 4.09 mmHg at month 12 (95% CI −7.64 to −0.55; *p* = 0.024) and 4.21 at month 24 (95% CI −7.95 to −0.48; *p* = 0.027)—as was mean arterial pressure (−3.53; 95% CI −5.69 to −1.38; *p* = 0.001, and −2.60; 95% CI −5.07 to −0.13; *p* = 0.039). The difference in diastolic pressure was confined to the first year (−3.35 at month 12; 95% CI −5.17 to −1.54; *p* < 0.001) and had gone by month 24 (−1.50; 95% CI −3.63 to +0.63; *p* = 0.17). Slopes were homogeneous throughout (all interaction *p* ≥ 0.10). The size and timing of these changes are what sodium–glucose cotransporter 2 inhibition would be expected to produce.

Peripheral oedema was equally common throughout, affecting 27.2% and 28.8% at entry and 21.8% and 22.4% at month 24, respectively (all *p* ≥ 0.70), with a slight fall in both cohorts that did not reach significance (McNemar test, adjusted *p* = 0.65 and *p* = 0.35). Neither volume depletion nor worsening fluid overload was apparent under dapagliflozin.

A fall of this size could in principle account for part of the antiproteinuric effect, so the covariance models were repeated with mean arterial pressure at baseline and month 24 added. The effect barely weakened: the adjusted GMR moved from 0.684 to 0.707 (95% CI 0.551–0.908; *p* = 0.007), and adjusting for systolic pressure instead gave 0.719 (95% CI 0.561–0.922; *p* = 0.010). In the subgroup on intensified immunosuppression the estimate changed little (0.371 to 0.376; 95% CI 0.234–0.607), and the chronic rate of eGFR decline was likewise almost unaffected (4.61 to 4.47 per year; 95% CI 1.64–7.31; *p* = 0.002).

The size of the association argues against blood pressure as the principal mechanism for the antiproteinuric effect. In the fully adjusted model, each 1 mmHg of mean arterial pressure at month 24 corresponded to a 1.3% difference in proteinuria (*p* = 0.024), so the 4 mmHg fall observed here would account for about 5%, against the 51.9% actually seen. The two also follow different time courses: blood pressure reached its nadir by month 12 and fell no further, whereas proteinuria went on declining through the second year. The correlation between the change in mean arterial pressure and the change in proteinuria was weak and virtually identical in the cohorts (Spearman r = 0.195, *p* = 0.018 and r = 0.193, *p* = 0.012, respectively), which is not what a mediator of the treatment effect would produce.

### 3.4. Tolerability and Persistence with Treatment

Persistence was assessed in all 301 patients who received dapagliflozin. Treatment was discontinued in eight (2.7%): five at the patient’s own request and three (1.0%) because of recurrent urinary tract infection. No other adverse event led to withdrawal, giving a persistence rate of 97.3%.

Among the 147 patients analysed, no adverse event attributable to dapagliflozin was recorded and none discontinued treatment. Given the retrospective design, this reflects the absence of documented events rather than active surveillance, and mild reactions managed outside the clinic are likely to have been under-recorded. The rate of discontinuation for urinary tract infection, however, matches that reported in the randomised trials, suggesting that serious events were reasonably well captured.

## 4. Discussion

Over two years, in 317 patients, most with biopsy-proven glomerular diseases, dapagliflozin was associated with a substantial and progressive fall in proteinuria and with a slower decline in kidney function during the second year. The pattern of the effect over time is more informative than its size, and three features stand out. Proteinuria kept falling into the second year rather than plateauing. Loss of eGFR was confined to the first year and had all but stopped by month 24, while in the historical cohort it accelerated. And the antiproteinuric effect was concentrated among patients whose disease was active enough to require intensified immunosuppression, a group that has been excluded from comparable studies.

The first of these features, the continuing fall in proteinuria, is also where our findings depart furthest from the randomised trials: the effect we observed was larger than that reported in these trials. DAPA-CKD found a 29.3% reduction in geometric mean albumin-to-creatinine ratio overall, but only 14.8% (95% CI 5.9–22.9) in participants without type 2 diabetes [9]; the clinical benefit in that subgroup was nonetheless clear [27]. We found 31.6% overall and 62.9% in the subgroup on intensified immunosuppression. Part of the difference comes from the measure itself: 24 h protein excretion and the albumin-to-creatinine ratio do not measure the same thing in glomerular disease. Another part comes from the population, defined here by biopsy rather than by an albuminuria threshold and more heterogeneous than a trial cohort, as routine practice is. Together they do not account for the whole gap. Since dapagliflozin became the standard of care as soon as it was introduced, a contemporaneous control group was not available; a comparison against historical controls, however, does not exclude residual confounding, and when an observational estimate exceeds the randomised evidence for the same drug, the true effect is probably smaller than the observational estimate.

The DIAMOND trial makes a different point. It randomised patients with non-diabetic proteinuric CKD, an eGFR above 25 mL/min/1.73 m^2^ and 24 h proteinuria of 500–3500 mg (close to our population) and found no effect on proteinuria at all, though the acute fall in measured GFR appeared as expected [14]. It ran for six weeks. Our data suggest why that may be too short: proteinuria fell by 33.8% in the first year and by a further 27.4% in the second, reaching 40.8% in the second year among patients on intensified immunosuppression. A six-week study cannot detect an effect that develops over months to years, so the null result of DIAMOND suggests that a longer treatment period is needed rather than casting doubt on whether the effect exists. A more recent crossover trial in non-diabetic stage 4 disease, with iohexol-measured GFR and 24 h proteinuria as co-primary outcomes, was similarly short [28].

Two observational series offer a closer comparison. The international cohort of the Immunonephrology Working Group of the European Renal Association and the Spanish GLOSEN group followed 493 patients with biopsy-proven glomerulonephritis and residual proteinuria of at least 1 g/day on renin–angiotensin blockade, reporting reductions of 35%, 41%, 45% and 48% at 3, 6, 9 and 12 months, consistent across the underlying diseases [29]. Our 33.8% at 12 months is of the same order, and the trajectory (a reduction that deepens rather than settling) is the same. A second series, confined to non-diabetic glomerulopathies and also measuring proteinuria in g/24 h, assessed it at 3–6 months and excluded patients on induction immunosuppression [16]. Our cohort complements both: it has a longer follow-up and, unlike the second series, it includes patients on induction immunosuppression.

The largest real-world experience with dapagliflozin points the same way. Across 93 Italian renal clinics, 1724 patients treated for a mean of four months showed a 25% fall in albuminuria, with a reduction of at least 30% in 48.3% [30]. Our responder rate in the second year was 59.2%. The two are not directly comparable: the Italian study had a much shorter exposure, measured albuminuria rather than 24 h proteinuria and defined its population by chronic kidney disease rather than by glomerular diagnosis, a distinction it could not make. Even so, the difference is in the direction expected of an effect that increases over time.

The second, the course of kidney function, is easier to reconcile with what is known, since it behaved as the pharmacology predicts. In the treated cohort, eGFR fell by 2.92 mL/min/1.73 m^2^ in the first year and then almost stopped falling, the change over the second year being −0.06 (95% CI −1.96 to +1.85). The first-year fall is compatible with the haemodynamic dip that follows restoration of tubuloglomerular feedback, but with annual measurements, and with a first-year decline no larger than in the historical cohort, we cannot show that this was its cause. The level of kidney function at month 24, however, did not differ significantly once baseline was taken into account (2.51 mL/min/1.73 m^2^, 95% CI −0.92 to 5.93; *p* = 0.15), so the slower second-year decline has yet to translate into a clearly higher eGFR; whether it does would need longer follow-up. In the historical cohort, the reverse held: the decline in eGFR accelerated to 5.09 mL/min/1.73 m^2^ in the second year. The chronic slope, between months 12 and 24, is the component most closely related to subsequent clinical events, and the case for using it rather than total decline rests on a meta-analysis of 47 randomised trials in more than 60,000 participants and on the workshops convened by the National Kidney Foundation with the Food and Drug Administration and the European Medicines Agency [31,32].

Again, our estimate exceeds the randomised evidence. DAPA-CKD reported a difference in eGFR chronic slope of 1.29 mL/min/1.73 m^2^ per year (95% CI 0.73–1.85) in patients without type 2 diabetes and 2.26 (1.88–2.64) in patients with diabetes, and a difference in total slope, over a median 2.4 years, of 0.95 (0.63–1.27) [8]; the prespecified analysis in focal segmental glomerulosclerosis gave 2.0 (0.6–3.5) [33]. EMPA-KIDNEY pointed the same way, its secondary analyses showing benefit on chronic slope across primary kidney diseases including glomerulonephritis [34,35]. We found 4.61 mL/min/1.73 m^2^ (1.82–7.41) for the chronic rate and 2.15 (0.44–3.87) for the total. The direction matches; the magnitude does not.

Two features could explain why the difference in the rate of eGFR decline between our dapagliflozin and historical cohorts exceeds that reported in the trials. The chronic slope in our study and that in DAPA-CKD are not measured over the same period. Ours covers the second year alone, the first interval that, with annual measurements, lies clearly beyond any acute haemodynamic effect. In DAPA-CKD, the chronic slope runs from the second week onwards and so includes most of the first year. The difference in chronic slope reported in DAPA-CKD is therefore smaller than ours. Both of our cohorts, moreover, include patients with active immune diseases, whom the randomised trials excluded; in these conditions, part of the early loss of function is potentially reversible, and dapagliflozin allows this recovery to be more complete, which may represent an effect additional to that of immunosuppression. We return to this below.

One result deserves separate comment. At month 24, a single patient on dapagliflozin had an eGFR below 15 mL/min/1.73 m^2^, against 20 of 170 in the historical cohort (0.7% versus 11.8%; *p* < 0.001). It needs to be read with some care. The dapagliflozin cohort started from a lower median eGFR, but the historical cohort had more patients close to the threshold—21 against 10 with a baseline eGFR below 25 mL/min/1.73 m^2^—and most of the controls who crossed it came from this group. Even so, the difference held among these patients: only one of the ten on dapagliflozin fell below 15 mL/min/1.73 m^2^, against 14 of the 21 controls. Rapid decline, defined as a loss of eGFR exceeding 5 mL/min/1.73 m^2^ per year, was likewise less common (27.2% versus 42.4%; *p* = 0.007).

The third feature is the least expected, and concerns patients whose disease was active at entry. Among them, month-24 proteinuria was 62.9% lower in the dapagliflozin than in the historical cohort at the same baseline value (adjusted GMR 0.371, 95% CI 0.234–0.586), whereas among the 219 patients from both cohorts who were not on intensified immunosuppression, no such difference was found (GMR 0.894, 95% CI 0.667–1.198; *p* = 0.45); the difference between the two subgroups was significant (*p* = 0.002). Restricted to the 164 patients who never received intensified immunosuppression during follow-up, the estimate was 0.754 (95% CI 0.554–1.027; *p* = 0.073), close to the effect reported in the randomised trials. Adding diagnostic category to the model barely changed the estimate (GMR 0.408), and the effect was similar across all diagnoses.

Published studies have approached these patients differently. The randomised trials excluded patients on high-dose immunosuppression altogether. The ERA–GLOSEN cohort excluded patients on induction treatment but kept 79 (16%) on maintenance immunosuppression and found the proportion reaching a 30% reduction in proteinuria to be identical in them and in those not receiving immunosuppression (*p* = 0.89) [29]. The series confined to non-diabetic glomerulopathies excluded anyone on prednisone above 20 mg daily, rituximab or cyclophosphamide within six months—our own threshold, used there as an exclusion criterion [16]. Some years ago, it was pointed out that background immunosuppression remains the unexamined variable in this field [17], and a recent review concluded that the benefit probably extends to such patients, though the data to show it are lacking [18]. Whether SGLT2 inhibitors belong in the routine management of glomerular disease, and on what evidence, has been debated directly between the groups working on it [36]. Two studies reached different conclusions and must be considered alongside ours. In a prospective cohort of 93 patients with biopsy-proven IgA nephropathy on full-dose renin–angiotensin blockade, SGLT2 inhibition reduced 24 h proteinuria by 22.9% at three months and 27.1% at six, independently of immunosuppressive use [37]. More directly, the ERA-GLOSEN investigators found that a serum albumin below 3.5 g/dL at initiation made a 30% reduction in proteinuria less likely (odds ratio 0.53, 95% CI 0.30–0.91) and concluded that patients with active disease are unlikely to respond [29]. This is the opposite of what we observed. Two small randomised trials in lupus nephritis, which, with the pilot trial of empagliflozin in glomerulonephritis [15], make up the randomised evidence in patients on background immunosuppression, define the remaining gap more precisely. In a crossover trial in 30 patients with inactive lupus nephritis on maintenance mycophenolate mofetil, six months of dapagliflozin reduced residual proteinuria by 36.1%, against 3.5% on standard care alone [21]. In a placebo-controlled trial in 79 patients with lupus nephritis on standard immunosuppression, twelve months of dapagliflozin produced a numerical but not significant reduction in proteinuria [22]. Neither was designed to examine patients whose disease required intensified treatment: the first excluded active nephritis, and the second, although some of its patients received cyclophosphamide, enrolled patients with long-standing disease and low proteinuria (median 0.4–0.6 g/24 h) and did not record disease activity systematically. Whether adding an SGLT2 inhibitor during intensified immunosuppression lowers proteinuria has therefore not yet been tested in a randomised trial, and it is to this question that our observations, with the caution they require, are relevant.

That conclusion, however, was not based on a prespecified criterion of disease activity: serum albumin was one of several candidate predictors in a regression model and was only afterwards interpreted as a marker of activity. Serum albumin reflects the nephrotic syndrome rather than immune activity. Many glomerulonephritides, including IgA nephropathy and lupus nephritis, are active with preserved albumin levels, and albumin levels also fall for reasons other than urinary protein loss, such as inflammation, malnutrition or liver disease. Our own marker, the intensity of immunosuppression, reflects something different: the clinical judgement that the disease required intensified treatment. It too, however, remains clinical rather than histological, and how closely clinical activity tracks morphological activity is not known.

A simpler explanation must be considered first. If the baseline visit fell at a different point in the treatment course in the two cohorts, with the historical patients assessed after immunosuppression had already taken effect and those on dapagliflozin assessed as it was starting, the difference would reflect the study design rather than the drug. The durations of immunosuppressive treatment extracted from the medical records show that this concern is justified: at baseline, patients on dapagliflozin had been treated for a median of 3 months (IQR 2–5) and the historical patients for 7 (IQR 3–12; *p* = 0.025), partly because some of the latter had received maintenance treatment for years. Several observations nonetheless argue that timing does not account for the difference. The historical subgroup was, if anything, the more active at entry: proteinuria of 3.5 g/24 h or more in 19.3% against 14.6%, serum albumin below 35 g/L in 21.1% against 9.8%, and much the same prevalence of oedema. Intensified immunosuppression was withdrawn at the same pace in both, with 66.7% and 65.9% still meeting the criterion at month 12. The estimate held when the analysis was confined to comparable baseline values (adjusted GMR 0.399 above 0.5 g/24 h, 0.369 above 1.0 g/24 h and 0.378 above 1.5 g/24 h) and when it was adjusted for the duration of immunosuppression and the agents used (0.341, 95% CI 0.196–0.594). Most tellingly, the effect was present not only in patients who had started immunosuppression within three months of baseline (0.263) but also in those treated for longer (0.502), in whom most of the response to it should already have occurred. The smaller estimate in the latter group, although not significantly different (interaction *p* = 0.17), suggests that part of the effect in the subgroup as a whole may still reflect timing.

Within the historical cohort, the subgroup on intensified immunosuppression fared worse than the remaining 113 patients of that cohort—a 4.6% fall against 29.8%—and of the 57, the 38 still on intensified treatment at month 12 did not improve at all, while the 19 in whom it had been reduced fell by 13.5%. Intensified treatment at entry therefore marks disease not yet controlled rather than disease about to remit, and the absence of spontaneous improvement is what one would expect.

A mechanism has nonetheless been proposed: beyond haemodynamics, SGLT2 inhibitors modulate inflammation, immunometabolism and oxidative stress and might thereby reduce the activity of glomerulonephritis itself—though the review setting this out described the evidence as speculative and awaiting clinical, histological and molecular confirmation [18]. Experimental work points the same way: in proteinuric non-diabetic nephropathy, dapagliflozin limited podocyte damage independently of any glycaemic effect [38]. Patients on intensified immunosuppression also begin with higher proteinuria, and adjustment for baseline is not randomisation; the finding is exploratory and needs confirming.

Before any of these findings can be attributed to dapagliflozin itself, one alternative explanation has to be set aside. Blood pressure fell further under dapagliflozin than in the historical cohort (systolic by 6.54 against 1.56 mmHg), a change whose magnitude and timing are typical of this drug class. It accounts, however, for very little of the effect on proteinuria. Adjusting for mean arterial pressure moved the estimate only from 0.684 to 0.707, and a 4 mmHg fall corresponds to about a 5% reduction in proteinuria, against the 51.9% observed. The timing differs too: blood pressure reached its lowest point at month 12 and went no further, while proteinuria kept falling. And where blood pressure did fall, proteinuria fell no more steeply under dapagliflozin than in the historical cohort, which would not be expected if the fall in blood pressure were responsible for the fall in proteinuria.

These findings have to be weighed against the limitations of this study, which follow chiefly from its design. Historical controls cannot simply be assumed comparable: concomitant care, referral patterns and thresholds for intervention all moved between 2014 and 2025. The cohorts were imbalanced at entry, most importantly in eGFR, and although multivariable adjustment and inverse probability of treatment weighting based on a propensity score address this, neither is a substitute for randomisation. The dapagliflozin cohort entered with more advanced disease, with lower eGFR and higher proteinuria, although the latter difference was not significant, and how this affects our estimates is hard to predict. More advanced disease usually progresses faster, which would count against dapagliflozin; values that start far from the mean, on the other hand, tend to move back towards it, which would favour dapagliflozin. Adjusting for the baseline of each outcome is meant to deal with the second problem, and our estimates changed little when baseline eGFR, age, diabetes, eGFR at 12 months, blood pressure and diagnostic category were added to the models. Some confounding by disease severity may nonetheless remain. The exclusions were not symmetrical either. Twenty patients in the historical cohort, but none in the dapagliflozin cohort, were excluded because they had reached end-stage kidney disease before month 24; as these were the patients whose disease progressed fastest, their exclusion makes the historical cohort appear better than it was and, if anything, understates the difference in kidney outcomes. Conversely, excluding the eight patients who stopped dapagliflozin and the seventeen who missed a visit or developed a malignancy may have favoured the dapagliflozin cohort, although together they made up less than a tenth of those treated. Proteinuria came from 24 h collections, which are prone to collection error and are not directly comparable with the albumin-to-creatinine ratio of the trials. Cystatin C was not measured, so eGFR was estimated from creatinine alone. Three time points cannot separate the acute and chronic components of the eGFR slope as cleanly as more frequent sampling would. Inclusion required three scheduled admissions, so patients who did not attend regularly are not represented. Forty-two patients had no histological diagnosis and were treated empirically, eleven of them in the subgroup on intensified immunosuppression. Follow-up was limited to 24 months because too few patients had reached a longer horizon by the end of the observation period. The subgroup analyses were exploratory and carry no multiplicity adjustment. Finally, our effect sizes exceed those of the randomised trials, and residual confounding is a more likely explanation for this than greater efficacy. The timing of immunosuppression was reconstructed from the cumulative duration of treatment recorded in the medical records rather than from exact dates, and these durations were inconsistent between visits in 15 patients. Patients on dapagliflozin had been treated for a shorter time at baseline, and although adjustment and stratification left the effect in place, a residual difference in timing may have inflated the apparent effect in the subgroup. Only a randomised trial can settle the question, and one designed for this population has recently been registered: a pilot, double-blind, placebo-controlled trial of dapagliflozin added to standard immunosuppression in 33 patients with active lupus nephritis, with the change in proteinuria over 12 weeks among its outcomes [39].

The strengths are the two-year horizon, the completeness of the data—proteinuria and eGFR were available for every patient at every visit, so nothing was imputed—the histological characterisation of most participants, all read by a single pathologist, and a single laboratory using one creatinine assay across both periods. Tolerability was good: among all 301 patients treated, persistence over two years was 97.3%, and the only withdrawals for an adverse event were three cases of recurrent urinary tract infection (Section 3.4).

## 5. Conclusions

In a two-year real-world cohort of patients with predominantly biopsy-proven glomerular diseases, dapagliflozin was associated with a progressive fall in proteinuria and with a slower decline in kidney function during the second year, although eGFR at two years did not differ significantly once baseline was taken into account. The benefit continued to accumulate during the second year rather than being limited to an initial response. It appeared concentrated among patients whose disease required intensified immunosuppression at entry—a group excluded from the trials and from the comparable observational literature—and was not explained by the accompanying fall in blood pressure. Because this study was observational and used historical controls, the true effects are likely to be smaller than those observed; however, their direction and timing are consistent with the randomised evidence [2,3,27] and with the observational experience in glomerular diseases [29], justifying testing dapagliflozin alongside intensified immunosuppression in a randomised trial.

## Figures and Tables

**Table 1:** Baseline characteristics of the dapagliflozin cohort and the historical cohort.

Variable	Dapagliflozin Cohort (N_1_ = 147)	Historical Cohort (N_2_ = 170)	*p*-Value
Age, years, median (IQR)	53.0 (43.0–63.0)	47.0 (38.8–57.3)	0.001
Female sex, *n* (%)	78 (53.1%)	88 (51.8%)	0.823
Biopsy-proven specific immune nephropathy ^1^, *n* (%)	114 (77.5%)	135 (79.4%)	0.784
IgAN	27 (18.4%)	18 (10.6%)	0.054
MN	14 (9.5%)	17 (10.0%)	1.000
FSGS	25 (17.0%)	20 (11.8%)	0.199
MCD	4 (2.7%)	9 (5.3%)	0.274
CIN	16 (10.9%)	13 (7.6%)	0.336
LN	11 (7.5%)	23 (13.5%)	0.102
MGN	13 (8.8%)	26 (15.3%)	0.089
MPGN	4 (2.7%)	9 (5.3%)	0.274
Diabetes mellitus ^2^, *n* (%)	40 (27.2%)	30 (17.6%)	0.043
Biopsy-proven diabetic nephropathy ^3^, *n* (%)	12 (8.2%)	4 (2.4%)	0.021
Hypertension, *n* (%)	134 (91.2%)	137 (80.6%)	0.010
Biopsy-proven hypertensive nephropathy ^4^, *n* (%)	38 (25.9%)	10 (5.9%)	<0.001
Intensified immunosuppression within the past 3 months, *n* (%)	41 (27.9%)	57 (33.5%)	0.330
ACEi or ARB use ^5^, *n* (%)	97 (65.9%)	119 (70.0%)	0.470
Loop diuretic use, *n* (%)	46 (31.3%)	46 (27.1%)	0.457
Thiazide diuretic use, *n* (%)	9 (6.1%)	14 (8.2%)	0.521
MRA use ^6^, *n* (%)	25 (17.0%)	32 (18.8%)	0.770
Systolic blood pressure, mmHg, median (IQR)	130 (120–145)	130 (120–140)	0.649
Diastolic blood pressure, mmHg, median (IQR)	80 (70–90)	80 (80–90)	0.247
Mean arterial pressure, mmHg, median (IQR)	98.3 (90.0–106.7)	96.7 (93.3–106.7)	0.875
Haemoglobin, g/L, median (IQR)	137 (123–147)	135 (123–151)	0.934
Haematocrit, L/L, median (IQR)	0.44 (0.39–0.48)	0.41 (0.38–0.46)	0.002
Serum creatinine ^7^, µmol/L, median (IQR)	125.0 (86.0–165.0)	95.5 (66.0–152.3)	0.004
eGFR, mL/min/1.73 m^2^, median (IQR)	50.0 (31.1–67.3)	67.0 (37.9–92.0)	0.001
G1 (≥90 mL/min/1.73 m^2^), *n* (%)	18 (12.2%)	49 (28.8%)	<0.001
G2 (60–89 mL/min/1.73 m^2^), *n* (%)	30 (20.4%)	43 (25.3%)	0.350
G3a (45–59 mL/min/1.73 m^2^), *n* (%)	31 (21.1%)	24 (14.1%)	0.137
G3b (30–44 mL/min/1.73 m^2^), *n* (%)	35 (23.9%)	23 (13.5%)	0.020
G4 (15–29 mL/min/1.73 m^2^), *n* (%)	33 (22.4%)	31 (18.3%)	0.401
G5 (<15 mL/min/1.73 m^2^), *n* (%)	0 (0.0%)	0 (0.0%)	-
Urea, mmol/L, median (IQR)	7.6 (5.9–11.0)	7.7 (5.2–10.8)	0.827
Uric acid, µmol/L, median (IQR)	345 (303–418)	369 (303–422)	0.478
Serum sodium, mmol/L, median (IQR)	141.0 (140.0–143.0)	141.0 (139.0–142.0)	0.176
Serum potassium, mmol/L, median (IQR)	4.6 (4.3–4.9)	4.7 (4.4–5.0)	0.05
Serum chloride, mmol/L, median (IQR)	107.0 (105.0–108.0)	104.0 (102.0–106.0)	<0.001
Fasting blood glucose, mmol/L, median (IQR)	5.41 (5.06–6.02)	5.16 (4.80–5.72)	0.003
Serum total protein, g/L, median (IQR)	70.0 (65.0–75.0)	70.0 (65.0–76.0)	0.888
Serum albumin, g/L, median (IQR)	41.0 (39.0–44.0)	42.0 (38.0–44.0)	0.464
Serum total cholesterol, mmol/L, median (IQR)	5.49 (4.67–6.36)	5.90 (5.13–7.13)	<0.001
Serum triglycerides, mmol/L, median (IQR)	1.60 (1.10–2.51)	1.41 (0.98–2.19)	0.159
Proteinuria, g/24 h, median (IQR)	0.95 (0.38–2.41)	0.62 (0.24–1.94)	0.078
<0.15 g/24 h, *n* (%)	12 (8.2%)	27 (15.9%)	0.041
0.15–0.49 g/24 h, *n* (%)	40 (27.2%)	41 (24.1%)	0.606
0.50–3.49 g/24 h, *n* (%)	76 (51.7%)	78 (45.9%)	0.313
≥3.50 g/24 h, *n* (%)	19 (12.9%)	24 (14.1%)	0.870

IgAN, immunoglobulin A nephropathy; MN, membranous nephropathy; FSGS, focal segmental glomerulosclerosis; MCD, minimal change disease; CIN, chronic interstitial nephritis; LN, lupus nephritis; MGN, mesangioproliferative glomerulonephritis; MPGN, membranoproliferative glomerulonephritis; IQR, interquartile range; eGFR, estimated glomerular filtration rate (CKD-EPI 2021 equation); ACEi, angiotensin-converting enzyme inhibitor; ARB, angiotensin II type 1 receptor blocker; MRA, mineralocorticoid receptor antagonist. ^1^ Histologically confirmed diffuse renal disease other than diabetic or hypertensive nephropathy. At baseline, these conditions did not necessarily require specific intensified immunosuppression. ^2^ Diagnosed according to laboratory criteria. ^3^ Histologically confirmed diabetic nephropathy, either coexisting with the specific (immune) nephropathy or as the principal cause of CKD. ^4^ Histologically confirmed hypertensive nephropathy, either coexisting with the specific (immune) nephropathy or as the principal cause of CKD. ^5^ ACEi or ARB were administered as standard of care at the highest dose tolerated according to the patient’s clinical status (blood pressure, serum potassium and stage of CKD). ^6^ Steroidal (spironolactone or eplerenone) or non-steroidal (finerenone). Finerenone was received by one patient in the dapagliflozin cohort and by no patient in the historical cohort. ^7^ Analytical methods for all laboratory variables are given in Section 2.2.

**Table 2:** Twenty-four-hour proteinuria over the 24-month observation period.

	Proteinuria, g/24 h, Median (IQR)	GMR vs. Baseline (95% CI)	Adjusted *p*-Value
**Dapagliflozin cohort (N_1_ = 147)**			
Baseline (Month 0)	0.95 (0.38–2.41)	-	-
Month 12	0.57 (0.23–1.95)	0.662 (0.560–0.781)	<0.001
Month 24	0.49 (0.19–1.19)	0.481 (0.399–0.579)	<0.001
Month 12 → Month 24	-	0.726 (0.629–0.839)	<0.001
**Historical cohort (N_2_ = 170)**			
Baseline (Month 0)	0.62 (0.24–1.94)	-	-
Month 12	0.56 (0.19–1.56)	0.777 (0.633–0.953)	0.013
Month 24	0.57 (0.18–1.74)	0.778 (0.638–0.948)	0.063
Month 12 → Month 24	-	1.002 (0.835–1.202)	>0.99
**Dapagliflozin vs. historical cohort**	Direct comparison, *p*	Adjusted GMR (95% CI)	*p*-value
Baseline (Month 0)	0.078	-	-
Month 12	0.464	0.940 (0.737–1.201)	0.621
Month 24	0.323	0.684 (0.534–0.878)	0.003

GMR, geometric mean ratio; CI, confidence interval; IQR, interquartile range. GMR values were derived from paired differences of log-transformed proteinuria. *p*-values are from the Wilcoxon signed-rank test on log-transformed values, Bonferroni-adjusted for three within-cohort comparisons. The final row of each block reports the change between months 12 and 24. In the last block, the two cohorts are compared at each visit directly, by the Mann–Whitney U test, and after adjustment for baseline, by analysis of covariance of log-transformed proteinuria on its log baseline value; these *p*-values are not adjusted for multiplicity.

**Table 3:** Proteinuria response at 24 months.

Outcome at Month 24	Dapagliflozin Cohort (N_1_ = 147), *n* (%)	Historical Cohort (N_2_ = 170), *n* (%)	OR (95% CI)
≥30% reduction	87 (59.2%)	65 (38.2%)	2.34 (1.49–3.68)
≥50% reduction	71 (48.3%)	44 (25.9%)	2.68 (1.67–4.29)
Partial remission	69 (46.9%)	44 (25.9%)	2.53 (1.58–4.06)
Complete remission (<0.3 g/24 h)	49 (33.3%)	69 (40.6%)	0.73 (0.46–1.16)
Progression (≥30% increase)	25 (17.0%)	52 (30.6%)	0.47 (0.27–0.80)

OR, odds ratio; CI, confidence interval. Partial remission was defined as proteinuria < 3.5 g/24 h combined with a reduction of at least 50% from baseline. Odds ratios are unadjusted; *p*-values were obtained using Fisher’s exact test.

**Table 4:** Proteinuria in patients receiving intensified immunosuppression at baseline.

	Proteinuria, g/24 h, Median (IQR)	GMR vs. Baseline (95% CI)	Adjusted *p*-Value
**Dapagliflozin cohort (n_1_ = 41)**			
Baseline (Month 0)	1.14 (0.28–2.35)	-	-
Month 12	0.57 (0.20–2.04)	0.548 (0.397–0.757)	0.002
Month 24	0.49 (0.11–1.12)	0.325 (0.226–0.467)	<0.001
Month 12 → Month 24	-	0.592 (0.446–0.785)	0.002
**Historical cohort (n_2_ = 57)**			
Baseline (Month 0)	0.59 (0.25–2.88)	-	-
Month 12	0.71 (0.20–2.15)	0.816 (0.562–1.185)	0.48
Month 24	0.93 (0.22–1.87)	0.954 (0.694–1.310)	>0.99
Month 12 → Month 24	-	1.168 (0.825–1.654)	>0.99

GMR, geometric mean ratio; CI, confidence interval; IQR, interquartile range. GMR values were derived from paired differences of log-transformed proteinuria. *p*-values are from the Wilcoxon signed-rank test on log-transformed values, Bonferroni-adjusted for three within-cohort comparisons. The final row of each block reports the change between months 12 and 24.

**Table 5:** Proteinuria response at 24 months in patients receiving intensified immunosuppression at baseline.

Outcome at Month 24	Dapagliflozin Cohort (n_1_ = 41), *n* (%)	Historical Cohort (n_2_ = 57), *n* (%)	OR (95% CI)
≥30% reduction	30 (73.2%)	18 (31.6%)	5.91 (2.43–14.37)
≥50% reduction	26 (63.4%)	14 (24.6%)	5.32 (2.22–12.79)
Partial remission	25 (61.0%)	14 (24.6%)	4.80 (2.01–11.46)
Complete remission (<0.3 g/24 h)	16 (39.0%)	20 (35.1%)	1.18 (0.52–2.72)
Progression (≥30% increase)	3 (7.3%)	21 (36.8%)	0.14 (0.04–0.49)

OR, odds ratio; CI, confidence interval. Partial remission was defined as proteinuria < 3.5 g/24 h combined with a reduction of at least 50% from baseline. Odds ratios are unadjusted; *p*-values were obtained with Fisher’s exact test.

**Table 6:** Histological diagnosis in patients receiving intensified immunosuppression at baseline, and 24-month change in proteinuria by diagnostic category.

Diagnostic Category	Dapagliflozin Cohort (n_1_ = 41), *n* (%)	Historical Cohort (n_2_ = 57), *n* (%)	GMR at Month 24, Dapagliflozin	GMR at Month 24, Historical	*p*-Value
Proliferative/immune-complex ^1^	18 (43.9%)	31 (54.4%)	0.411	1.047	0.002
Podocytopathy ^2^	15 (36.6%)	13 (22.8%)	0.214	0.701	0.010
Other ^3^	6 (14.6%)	4 (7.0%)	0.406	0.751	0.76
No biopsy performed ^4^	2 (4.9%)	9 (15.8%)	-	-	-

GMR, geometric mean ratio, expressed relative to the individual baseline value; *p*-values are from the Mann–Whitney U test on log-transformed changes. Where more than one histological diagnosis was recorded, patients were assigned to the category of the leading immune nephropathy. ^1^ IgA nephropathy, lupus nephritis, membranoproliferative glomerulonephritis, mesangioproliferative glomerulonephritis. ^2^ Minimal change disease, focal segmental glomerulosclerosis, membranous nephropathy. ^3^ Chronic interstitial nephritis, hypertensive nephropathy. ^4^ Patients treated empirically without histological confirmation; excluded from the analyses by diagnostic category.

**Table 7:** Estimated glomerular filtration rate over the 24-month observation period.

	eGFR, mL/min/1.73 m^2^, Median (IQR)	Change from Previous Value, Mean (95% CI)	Change from Previous Value, Median (IQR)	Adjusted *p*-Value
**Dapagliflozin cohort (N_1_ = 147)**				
Baseline (Month 0)	50.0 (31.1–67.3)	-	-	-
Month 12	46.3 (31.1–64.0)	−2.92 (−4.90 to −0.94)	−3.10 (−8.60 to +2.80)	0.004
Month 24	44.0 (32.4–66.1)	−0.06 (−1.96 to +1.85)	0.00 (−5.70 to +5.50)	>0.99
Month 0 → Month 24	-	−2.98 (−5.25 to −0.70)	−2.90 (−11.40 to +4.20)	0.011
**Historical cohort (N_2_ = 170)**				
Baseline (Month 0)	67.0 (37.9–92.0)	-	-	-
Month 12	62.2 (35.1–93.4)	−2.19 (−4.81 to +0.43)	−2.00 (−12.05 to +7.25)	0.36
Month 24	60.0 (28.8–85.3)	−5.09 (−7.05 to −3.14)	−4.70 (−11.35 to +3.25)	<0.001
Month 0 → Month 24	-	−7.28 (−9.86 to −4.70)	−7.55 (−17.00 to +1.00)	<0.001

IQR, interquartile range; CI, confidence interval. Changes are given both as means with 95% confidence intervals and as medians with interquartile ranges. The mean is reported because the rate of change in eGFR is an additive quantity and is the form in which slopes are conventionally expressed, and because the covariance models below estimate adjusted means; the median is given alongside it because the distribution of individual changes departed from normality (Shapiro–Wilk test *p* = 0.002 and *p* < 0.001 for the dapagliflozin and historical cohorts, respectively). The two summaries agree closely at every interval. *p*-values are from the Wilcoxon signed-rank test, Bonferroni-adjusted for three within-cohort comparisons. Friedman test across the three visits: χ^2^ = 13.5, *p* = 0.001 for the dapagliflozin cohort and χ^2^ = 41.0, *p* < 0.001 for the historical cohort. The final row of each block reports the cumulative change from baseline to month 24.

**Table 8:** Kidney outcomes at 24 months.

Outcome at Month 24	Dapagliflozin Cohort (N_1_ = 147), *n* (%)	Historical Cohort (N_2_ = 170), *n* (%)	OR (95% CI)
≥40% decline in eGFR	8 (5.4%)	24 (14.1%)	0.35 (0.15–0.81)
≥30% decline in eGFR	19 (12.9%)	35 (20.6%)	0.57 (0.31–1.05)
eGFR <15 mL/min/1.73 m^2^	1 (0.7%)	20 (11.8%)	0.05 (0.01–0.39)
Rapid decline (>5 mL/min/1.73 m^2^ per year)	40 (27.2%)	72 (42.4%)	0.51 (0.32–0.81)
Decline >10%	66 (44.9%)	97 (57.1%)	0.61 (0.39–0.96)
Stable (−10% to +10%)	44 (29.9%)	46 (27.1%)	1.15 (0.71–1.88)
Improvement >10%	37 (25.2%)	27 (15.9%)	1.78 (1.02–3.10)

OR, odds ratio; CI, confidence interval. The first four rows describe progression and are not mutually exclusive. Rapid decline was defined, in accordance with current guidance, as a sustained loss of more than 5 mL/min/1.73 m^2^ per year. The final three rows form an exhaustive and mutually exclusive classification of the 24-month trajectory; a change of up to 10% in either direction was taken as stability, since biological variation in creatinine generation and clearance together with analytical variation in the assay may account for a change of this magnitude. The distribution across these three categories did not quite reach significance (χ^2^ test, *p* = 0.053). Odds ratios are unadjusted; *p*-values were obtained with Fisher’s exact test.

**Table 9:** Estimated glomerular filtration rate (eGFR) in patients receiving intensified immunosuppression at baseline.

	eGFR, mL/min/1.73 m^2^, Median (IQR)	Change from Previous Value, Mean (95% CI)	Change from Previous Value, Median	Adjusted *p*-Value
**Dapagliflozin cohort (n_1_ = 41)**				
Baseline (Month 0)	62.0 (37.0–90.6)	-	-	-
Month 12	54.9 (35.2–82.9)	−4.68 (−8.63 to −0.72)	−3.40	0.042
Month 24	70.4 (34.0–81.1)	+0.29 (−3.87 to +4.44)	0.00	>0.99
Month 0 → Month 24	-	−4.39 (−9.71 to +0.93)	−6.90	0.10
**Historical cohort (n_2_ = 57)**				
Baseline (Month 0)	82.1 (44.0–104.0)	-	-	-
Month 12	70.1 (30.5–108.5)	−3.04 (−8.28 to +2.19)	−2.00	>0.99
Month 24	71.2 (27.5–90.5)	−6.62 (−10.83 to −2.41)	−6.00	0.013
Month 0 → Month 24	-	−9.67 (−15.03 to −4.31)	−10.10	0.002

IQR, interquartile range; CI, confidence interval. Changes are given from the preceding visit except in the final row of each block, which reports the cumulative change from baseline to month 24. The *p*-values are from the Wilcoxon signed-rank test, Bonferroni-adjusted for three within-cohort comparisons. Medians of the distribution and medians of the individual changes are distinct quantities: the former may shift although the latter is zero, since different patients occupy the middle rank at each visit.

**Table 10:** Blood pressure and peripheral oedema over the 24-month observation period.

	Dapagliflozin Cohort (D) (N_1_ = 147)	Historical Cohort (H) (N_2_ = 170)	Mean Change from Baseline, D vs. H (Within-Cohort *p*)	Between-Cohort *p*
**SBP, mmHg,** **median (IQR)**				
Baseline (Month 0)	130 (120–145)	130 (120–140)	-	-
Month 12	130 (120–140)	130 (120–140)	−6.31 (*p* < 0.001) vs. −1.59 (*p* > 0.99)	0.024
Month 24	130 (120–140)	130 (120–140)	−6.54 (*p* = 0.002) vs. −1.56 (*p* = 0.90)	0.027
**DBP, mmHg,** **median (IQR)**				
Baseline (Month 0)	80 (70–90)	80 (80–90)	-	-
Month 12	80 (70–80)	80 (80–90)	−3.57 (*p* = 0.001) vs. −0.59 (*p* > 0.99)	<0.001
Month 24	80 (75–80)	80 (80–90)	−2.07 (*p* = 0.22) vs. −0.98 (*p* > 0.99)	0.17
**MAP, mmHg,** **median (IQR)**				
Baseline (Month 0)	98.3 (90.0–106.7)	96.7 (93.3–106.7)	-	-
Month 12	95.0 (90.0–100.0)	96.7 (93.3–103.8)	−4.41 (*p* < 0.001) vs. −0.88 (*p* = 0.89)	0.001
Month 24	95.0 (91.7–100.0)	96.7 (93.3–106.7)	−3.77 (*p* = 0.017) vs. −1.18 (*p* = 0.67)	0.039
**Peripheral oedema,** ***n* (%)**				
Baseline (Month 0)	40 (27.2%)	49 (28.8%)	-	0.80
Month 12	36 (24.5%)	45 (26.5%)	-	0.70
Month 24	32 (21.8%)	38 (22.4%)	-	>0.99

SBP, systolic blood pressure; DBP, diastolic blood pressure; MAP, mean arterial pressure. Blood pressure values are medians with interquartile ranges; oedema is given as counts and percentages. Mean changes from baseline are in mmHg, shown for the dapagliflozin and historical cohorts, respectively, each with its within-cohort *p*-value from the Wilcoxon signed-rank test, Bonferroni-adjusted for three comparisons. Friedman test across the three visits in the dapagliflozin cohort: χ^2^ = 16.6 (*p* < 0.001) for SBP, χ^2^ = 13.2 (*p* = 0.001) for DBP and χ^2^ = 15.4 (*p* < 0.001) for MAP; in the historical cohort, all *p* ≥ 0.28. The between-cohort *p*-value is from an analysis of covariance on the value at that visit, adjusted for the corresponding baseline value; for oedema, it is from Fisher’s exact test comparing the cohorts at that visit.

## Data Availability

The anonymised data supporting the findings of this study are available from the corresponding author upon reasonable request. The data are not publicly available because of restrictions relating to patient privacy.

## Supplementary Materials

The following supporting information can be downloaded at: https://www.mdpi.com/article/10.3390/biomedicines14102353/s1. Figure S1: Flow of participants through the study. Patients were eligible for analysis if they had complete assessments at baseline and at months 12 and 24, each within one month; subgroups were defined by intensified immunosuppression at baseline; Table S1: Effect of dapagliflozin under alternative adjustment and weighting. (A) All outcomes; (B) sensitivity analyses in the subgroup on intensified immunosuppression; Table S2: Immunosuppressive treatment at baseline in the subgroup on intensified immunosuppression.

## References

[1] GBD 2023 Chronic Kidney Disease Collaborators (2025). Global, regional, and national burden of chronic kidney disease in adults, 1990–2023, and its attributable risk factors: A systematic analysis for the Global Burden of Disease Study 2023. Lancet.

[2] Heerspink H.J.L., Stefánsson B.V., Correa-Rotter R., Chertow G.M., Greene T., Hou F.-F., Mann J.F.E., McMurray J.J.V., Lindberg M., Rossing P. (2020). Dapagliflozin in patients with chronic kidney disease. N. Engl. J. Med..

[3] The EMPA-KIDNEY Collaborative Group (2023). Empagliflozin in patients with chronic kidney disease. N. Engl. J. Med..

[4] Nuffield Department of Population Health Renal Studies Group, SGLT2 Inhibitor Meta-Analysis Cardio-Renal Trialists’ Consortium (2022). Impact of diabetes on the effects of sodium glucose co-transporter-2 inhibitors on kidney outcomes: Collaborative meta-analysis of large placebo-controlled trials. Lancet.

[5] Kidney Disease: Improving Global Outcomes (KDIGO) CKD Work Group (2024). KDIGO 2024 Clinical Practice Guideline for the Evaluation and Management of Chronic Kidney Disease. Kidney Int..

[6] Vallon V., Thomson S.C. (2017). Targeting renal glucose reabsorption to treat hyperglycaemia: The pleiotropic effects of SGLT2 inhibition. Diabetologia.

[7] Yau K., Dharia A., Alrowiyti I., Cherney D.Z.I. (2024). SGLT2 inhibitors and kidney protection: Mechanisms beyond tubuloglomerular feedback. Kidney360.

[8] Heerspink H.J.L., Jongs N., Chertow G.M., Langkilde A.M., McMurray J.J.V., Correa-Rotter R., Rossing P., Sjöström C.D., Stefansson B.V., Toto R.D. (2021). Effect of dapagliflozin on the rate of decline in kidney function in patients with chronic kidney disease with and without type 2 diabetes: A prespecified analysis from the DAPA-CKD trial. Lancet Diabetes Endocrinol..

[9] Jongs N., Greene T., Chertow G.M., McMurray J.J.V., Langkilde A.M., Correa-Rotter R., Rossing P., Sjöström C.D., Stefansson B.V., Toto R.D. (2021). Effect of dapagliflozin on urinary albumin excretion in patients with chronic kidney disease with and without type 2 diabetes: A prespecified analysis from the DAPA-CKD trial. Lancet Diabetes Endocrinol..

[10] Wheeler D.C., Toto R.D., Stefánsson B.V., Jongs N., Chertow G.M., Greene T., Hou F.-F., McMurray J.J.V., Correa-Rotter R., Rossing P. (2021). A pre-specified analysis of the DAPA-CKD trial demonstrates the effects of dapagliflozin on major adverse kidney events in patients with IgA nephropathy. Kidney Int..

[11] Kidney Disease: Improving Global Outcomes (KDIGO) Glomerular Diseases Work Group (2021). KDIGO 2021 Clinical Practice Guideline for the Management of Glomerular Diseases. Kidney Int..

[12] Kidney Disease: Improving Global Outcomes (KDIGO) IgAN and IgAV Work Group (2025). KDIGO 2025 Clinical Practice Guideline for the Management of Immunoglobulin A Nephropathy (IgAN) and Immunoglobulin A Vasculitis (IgAV). Kidney Int..

[13] Kidney Disease: Improving Global Outcomes (KDIGO) Lupus Nephritis Work Group (2024). KDIGO 2024 Clinical Practice Guideline for the Management of Lupus Nephritis. Kidney Int..

[14] Cherney D.Z.I., Dekkers C.C.J., Barbour S.J., Cattran D., Abdul Gafor A.H., Greasley P.J., Laverman G.D., Lim S.K., Di Tanna G.L., Reich H.N. (2020). Effects of the SGLT2 inhibitor dapagliflozin on proteinuria in non-diabetic patients with chronic kidney disease (DIAMOND): A randomised, double-blind, crossover trial. Lancet Diabetes Endocrinol..

[15] Hammad H., Shaaban A., Philips M.V., Fayed A., Abdelaziz T.S. (2023). Effect of sodium–glucose transporter 2 inhibitor empagliflozin on proteinuria and kidney function progression in patients with non-diabetic glomerulonephritis: A pilot superiority randomized controlled trial. Int. Urol. Nephrol..

[16] Vargas-Brochero M.J., Russo I., Mazzierli T., Cara A., Berti G.M., Milheiro J., Thongprayoon C., Allinovi M., Somma C., Raglianti V. (2026). Antiproteinuric effect of SGLT2 inhibitors in non-diabetic glomerulopathies is dependent on body mass index. Nephrol. Dial. Transplant..

[17] Patoulias D., Popovic D.S. (2023). Are SGLT-2 inhibitors “the elephant in the room” of non-diabetic glomerulonephritis? Considerations about the background immunosuppressive treatment. Int. Urol. Nephrol..

[18] Del Vecchio L., Peiti S., Pucci Bella G., Locatelli F. (2025). SGLT2 inhibitors in glomerulonephritis: Beyond nephroprotection?. J. Clin. Med..

[19] Ferro C.J., Wanner C., Luyckx V., Fontana M., Gallego D., Vanholder R., Torra R., Ortiz A., Stronger Kidneys Taskforce of the European Renal Association, the European Kidney Health Alliance and the European Kidney Patients Federation (2025). A call for urgent action on chronic kidney disease across Europe. Lancet Reg. Health Eur..

[20] Mitić I., Laganović M., Marinova I., Gancheva N., Nakić V., Melentijevic D., Paskalev E., Vajd R., Škoberne A. (2022). Chronic kidney disease in Balkan countries—A call to action for timely diagnosis and monitoring. Diagnostics.

[21] Vajgel G., da Silva Júnior B.M., Silva Miranda Filho C.R., Barbosa Lyra de Oliveira C., do Nascimento Costa D.M., Dantas de Lima C.A., Valente L.M., Sandrin-Garcia P. (2025). Efficacy and safety of dapagliflozin in inactive lupus nephritis: A randomized crossover trial. Nephrol. Dial. Transplant..

[22] Mohamed N., Zayed K.N., Elhadedy M.A., Badawi M.H., Mortada W.I., Nabieh K.A., Sobh M.A., Refaie A.F. (2026). Dapagliflozin in lupus nephritis: Renal and hematologic outcomes from a randomized controlled trial. Clin. Kidney J..

[23] Kidney Disease: Improving Global Outcomes (KDIGO) Blood Pressure Work Group (2021). KDIGO 2021 Clinical Practice Guideline for the Management of Blood Pressure in Chronic Kidney Disease. Kidney Int..

[24] Inker L.A., Eneanya N.D., Coresh J., Tighiouart H., Wang D., Sang Y., Crews D.C., Doria A., Estrella M.M., Froissart M. (2021). New creatinine- and cystatin C-based equations to estimate GFR without race. N. Engl. J. Med..

[25] Kidney Disease: Improving Global Outcomes (KDIGO) Glomerulonephritis Work Group (2012). KDIGO Clinical Practice Guideline for Glomerulonephritis. Kidney Int. Suppl..

[26] von Elm E., Altman D.G., Egger M., Pocock S.J., Gøtzsche P.C., Vandenbroucke J.P., STROBE Initiative (2007). The Strengthening the Reporting of Observational Studies in Epidemiology (STROBE) statement: Guidelines for reporting observational studies. Lancet.

[27] Wheeler D.C., Stefánsson B.V., Jongs N., Chertow G.M., Greene T., Hou F.-F., McMurray J.J.V., Correa-Rotter R., Rossing P., Toto R.D. (2021). Effects of dapagliflozin on major adverse kidney and cardiovascular events in patients with diabetic and non-diabetic chronic kidney disease: A prespecified analysis from the DAPA-CKD trial. Lancet Diabetes Endocrinol..

[28] Trillini M., Villa A., Perna A., Peracchi T., Fidone D., Rubis N., Guarinoni C., Martinetti D., Chiappa A., Gamba T. (2025). Randomized trial of dapagliflozin in patients with nondiabetic stage 4 CKD. Kidney Int. Rep..

[29] Caravaca-Fontán F., Stevens K., Padrón M., Huerta A., Montomoli M., Villa J., González F., Vega C., López Mendoza M., Fernández L. (2024). Sodium-glucose cotransporter 2 inhibition in primary and secondary glomerulonephritis. Nephrol. Dial. Transplant..

[30] Minutolo R., Borrelli S., Ambrosini A., Amoroso L., Aucella F., Batini V., Battaglia Y., Bregoli L., Cantaluppi V., Cianciolo G. (2025). Efficacy and safety of dapagliflozin in patients with CKD: Real-world experience in 93 Italian renal clinics. Clin. Kidney J..

[31] Inker L.A., Heerspink H.J.L., Tighiouart H., Levey A.S., Coresh J., Gansevoort R.T., Simon A.L., Ying J., Beck G.J., Wanner C. (2019). GFR slope as a surrogate end point for kidney disease progression in clinical trials: A meta-analysis of treatment effects of randomized controlled trials. J. Am. Soc. Nephrol..

[32] Levey A.S., Gansevoort R.T., Coresh J., Inker L.A., Heerspink H.J.L., Grams M.E., Greene T., Tighiouart H., Matsushita K., Ballew S.H. (2020). Change in albuminuria and GFR as end points for clinical trials in early stages of CKD: A scientific workshop sponsored by the National Kidney Foundation in collaboration with the US Food and Drug Administration and European Medicines Agency. Am. J. Kidney Dis..

[33] Wheeler D.C., Jongs N., Stefansson B.V., Chertow G.M., Greene T., Hou F.F., Langkilde A.M., McMurray J.J.V., Rossing P., Nowicki M. (2022). Safety and efficacy of dapagliflozin in patients with focal segmental glomerulosclerosis: A prespecified analysis of the dapagliflozin and prevention of adverse outcomes in chronic kidney disease (DAPA-CKD) trial. Nephrol. Dial. Transplant..

[34] The EMPA-KIDNEY Collaborative Group (2024). Impact of primary kidney disease on the effects of empagliflozin in patients with chronic kidney disease: Secondary analyses of the EMPA-KIDNEY trial. Lancet Diabetes Endocrinol..

[35] The EMPA-KIDNEY Collaborative Group (2024). Effects of empagliflozin on progression of chronic kidney disease: A prespecified secondary analysis from the EMPA-KIDNEY trial. Lancet Diabetes Endocrinol..

[36] Caravaca-Fontán F., Del Vecchio L., Praga M., Floege J., Zoccali C. (2024). Sodium glucose co-transporter 2 inhibitors in the treatment of glomerular diseases: A CKJ controversy. Clin. Kidney J..

[37] Dong Y., Shi S., Liu L., Zhou X., Lv J., Zhang H. (2023). Effect of SGLT2 inhibitors on the proteinuria reduction in patients with IgA nephropathy. Front. Med..

[38] Cassis P., Locatelli M., Cerullo D., Corna D., Buelli S., Zanchi C., Villa S., Morigi M., Remuzzi G., Benigni A. (2018). SGLT2 inhibitor dapagliflozin limits podocyte damage in proteinuric nondiabetic nephropathy. JCI Insight.

[39] ClinicalTrials.gov Dapagliflozin in Active Lupus Nephritis (Dapa-Active LN); NCT07323524. NCT07323524.

