# Supplementary material for: Real-World Renal Effects of Dapagliflozin in Bulgarian Patients with Chronic Kidney Disease of Predominantly Glomerular Origin: A Two-Year Comparative Cohort Study with Historical Controls
Source: Biomedicines. 2026 Oct 16;14(10):2353. doi: 10.3390/biomedicines14102353 (PMC13701937; doi:10.3390/biomedicines14102353)
Supplement: Supplementary file 1 [file biomedicines-14-02353-s001.zip › biomedicines-4586716-supplementary.pdf]

Supplementary Materials

Real-World Renal Effects of Dapagliflozin in Bulgarian Patients with Chronic Kidney Disease of Predominantly Glomerular Origin: A Two-Year Comparative Cohort Study with Historical Controls

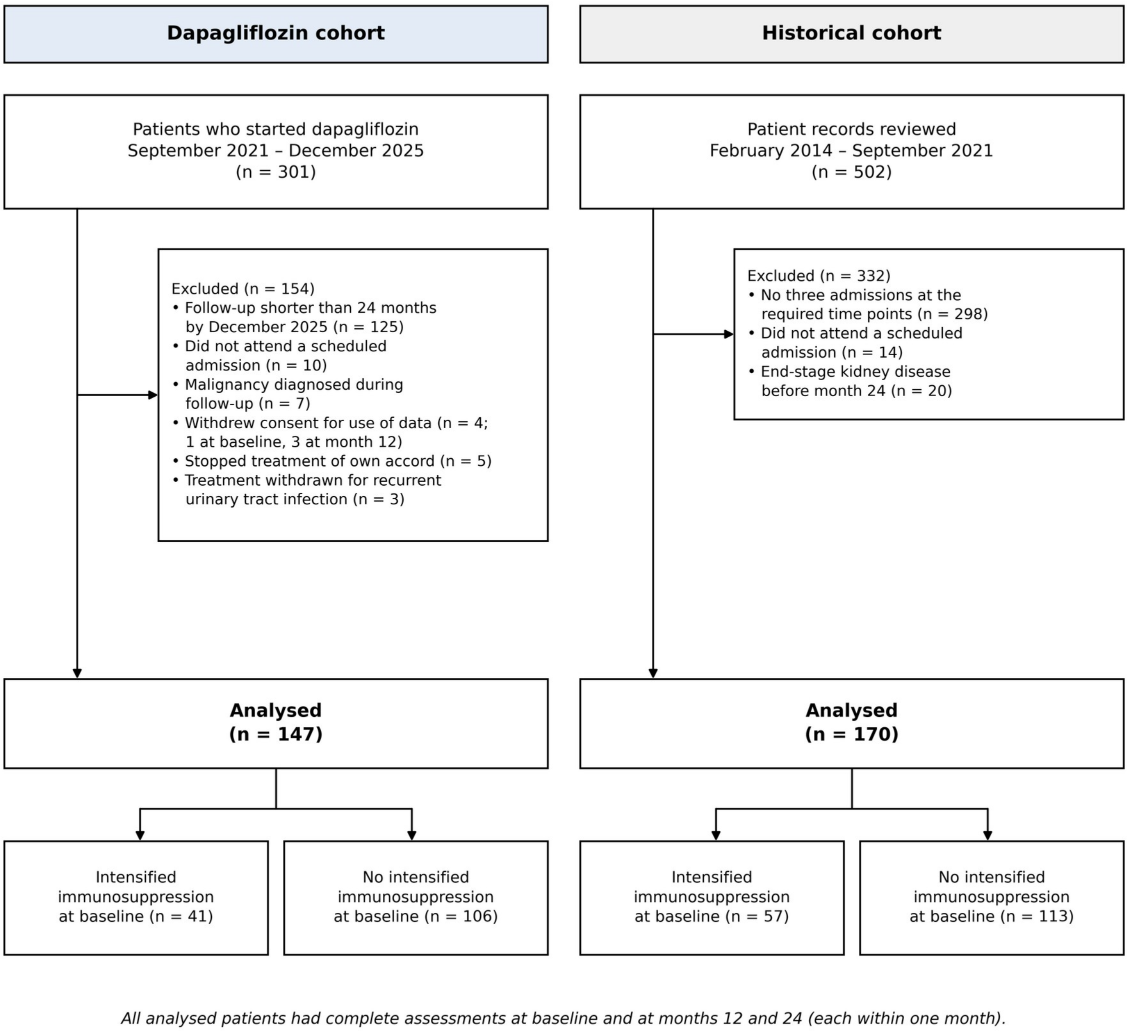

Figure S1. Flow of participants through the study. Patients were eligible for analysis if they had complete assessments at baseline and at months 12 and 24, each within one month; subgroups were defined by intensified immunosuppression at baseline.

Table S1. Effect of dapagliflozin under alternative adjustment and weighting. (A) All outcomes; (B) sensitivity analyses in the subgroup on intensified immunosuppression.

(A)

Outcome	Principal model	Partially adjusted	Fully adjusted	IPTW
Proteinuria at month 24, all patients (GMR)	0.684 (0.534–0.878) p = 0.003	0.679 (0.527–0.876) p = 0.003	0.664 (0.514–0.857) p = 0.002	0.616 (0.465–0.816) p < 0.001
Proteinuria, intensified immunosuppression (GMR)	0.371 (0.234–0.586) p < 0.001	0.407 (0.252–0.657) p < 0.001	0.335 (0.198–0.566) p < 0.001	0.350 (0.215–0.570) p < 0.001
Proteinuria, no intensified immunosuppression (GMR)	0.894 (0.667–1.198) p = 0.451	0.832 (0.616–1.123) p = 0.228	0.905 (0.669–1.224) p = 0.516	0.788 (0.561–1.107) p = 0.168
Chronic eGFR slope, months 12–24 (difference)	4.61 (1.82–7.41) p = 0.001	4.73 (1.89–7.57) p = 0.001	4.92 (1.97–7.86) p = 0.001	5.43 (2.30–8.56) p < 0.001
eGFR at month 24 (difference)	2.51 (−0.92 to 5.93) p = 0.151	3.54 (0.14–6.94) p = 0.041	2.79 (−0.72 to 6.30) p = 0.119	3.70 (0.01–7.40) p = 0.050

Values are estimates (95% CI) and p-values for dapagliflozin against the historical cohort. GMR, geometric mean ratio of 24-hour proteinuria at month 24; eGFR differences in mL/min/1.73 m^2 (per year for the chronic slope). Principal model: adjusted for the baseline value of the outcome. Partially adjusted: additionally for baseline eGFR (proteinuria models) or baseline proteinuria (eGFR models), age and diabetes. Fully adjusted: additionally for sex, hypertension, diagnostic category, renin–angiotensin system blockade, mineralocorticoid receptor antagonists, loop and thiazide diuretics, baseline mean arterial pressure and, in whole-cohort models, intensified immunosuppression at entry. IPTW: inverse probability of treatment weighting based on a propensity score from the same baseline variables together with baseline eGFR and proteinuria, with robust standard errors; after weighting, all standardised differences were below 0.05. Confidence intervals are based on the t distribution.

(B)

Model, subgroup on intensified immunosuppression (n = 98)	GMR (95% CI)
Principal model (baseline proteinuria)	0.371 (0.234–0.586), p < 0.001
+ duration of immunosuppression before baseline	0.384 (0.240–0.614), p < 0.001
+ duration + cyclophosphamide and azathioprine use	0.364 (0.223–0.591), p < 0.001
Fully adjusted + duration + agents	0.341 (0.196–0.594), p < 0.001
Same, excluding 5 patients with uncertain baseline duration	0.363 (0.201–0.654), p = 0.001
Fully adjusted + immunosuppressive status at months 12 and 24	0.326 (0.191–0.554), p < 0.001
Treated ≤3 months before baseline (n = 42)	0.263 (0.110–0.625), p = 0.003
Treated >3 months before baseline (n = 56)	0.502 (0.298–0.845), p = 0.010
Interaction, dapagliflozin × duration stratum	p = 0.17

Duration of immunosuppression is the longest cumulative duration of any agent up to baseline, log-transformed in the models. Fully adjusted: covariates as in (A), together with baseline eGFR.

Table S2. Immunosuppressive treatment at baseline in the subgroup on intensified immunosuppression.

Variable	Dapagliflozin (n = 41)	Historical (n = 57)	p-value
Agents received, n **(%)**			
Corticosteroid	37 (90.2)	45 (78.9)	0.172
Cyclophosphamide	19 (46.3)	17 (29.8)	0.137
Azathioprine	2 (4.9)	13 (22.8)	0.021
Mycophenolate mofetil	4 (9.8)	5 (8.8)	1.000
Ciclosporin	0	4 (7.0)	0.137
Intravenous immunoglobulin	0	0	–
Two or more agents	19 (46.3)	24 (42.1)	0.686
Duration of immunosuppression before baseline			
Cumulative duration, months, median (IQR)	3 (2–5)	7 (3–12)	0.025
≤3 months, n (%)	21 (51.2)	21 (36.8)	0.214
>6 months, n (%)	9 (22.0)	31 (54.4)	0.002
Corticosteroid duration in users, months, median (IQR)	3 (2–5)	7 (3–12)	0.066

Intensified immunosuppression was defined as treatment at any point in the three months before baseline with a corticosteroid at a dose equivalent to at least 20 mg prednisolone daily, or with azathioprine, mycophenolate mofetil, cyclophosphamide, ciclosporin or intravenous immunoglobulin at standard doses, alone or in combination. Durations are cumulative months of treatment up to baseline, extracted retrospectively from the medical records; the cumulative duration is that of the longest-used agent. Categorical variables were compared by Fisher's exact test and durations by the Mann–Whitney U test.
